# Systematic evaluation of multiple qPCR platforms, NanoString and miRNA-Seq for microRNA biomarker discovery in human biofluids

**DOI:** 10.1038/s41598-021-83365-z

**Published:** 2021-02-24

**Authors:** Lewis Z. Hong, Lihan Zhou, Ruiyang Zou, Chin Meng Khoo, Adeline Lai San Chew, Chih-Liang Chin, Shian-Jiun Shih

**Affiliations:** 1 Translational Biomarkers, MSD, Singapore, Singapore; 2 MiRXES Lab, Singapore, Singapore; 3 Yong Loo Lin School of Medicine, National University of Singapore, Singapore, Singapore; 4 Department of Medicine, National University Hospital, Singapore, Singapore

**Keywords:** Biochemistry, RNA, Biological techniques, Gene expression analysis, Genomic analysis

## Abstract

Aberrant miRNA expression has been associated with many diseases, and extracellular miRNAs that circulate in the bloodstream are remarkably stable. Recently, there has been growing interest in identifying cell-free circulating miRNAs that can serve as non-invasive biomarkers for early detection of disease or selection of treatment options. However, quantifying miRNA levels in biofluids is technically challenging due to their low abundance. Using reference samples, we performed a cross-platform evaluation in which miRNA profiling was performed on four different qPCR platforms (MiRXES, Qiagen, Applied Biosystems, Exiqon), nCounter technology (NanoString), and miRNA-Seq. Overall, our results suggest that using miRNA-Seq for discovery and targeted qPCR for validation is a rational strategy for miRNA biomarker development in clinical samples that involve limited amounts of biofluids.

## Introduction

Since their discovery more than two decades ago, microRNAs (miRNAs) have been recognized for their critical roles in gene regulation^1, 2^. miRNAs are small RNA molecules (~ 22 nucleotides in size) that exert their effects by suppressing the translation or inducing the degradation of their target mRNAs^3, 4^, and play important roles in a wide range of biologic and pathologic processes^5, 6^. Due to their tissue specificity and unusually high stability in biofluids, circulating cell-free miRNAs have emerged as a promising class of non-invasive biomarkers for human disease^7–10^. MiRNA expression profiling in plasma and serum has the potential for identifying miRNA biomarkers that are informative for early disease diagnosis and to predict response to therapy^11–14^. However, obtaining expression profiles of miRNAs in biofluids is technically challenging due to their low abundance, the small size of mature miRNAs, and the high degree of sequence similarity between family members^15^.

Many platforms have been developed for quantitating miRNA expression; they are typically based on quantitative PCR (qPCR), hybridization, or next-generation sequencing technology^16–18^. Previous studies have compared the performance characteristics of different miRNA profiling methods^15–17^ and platform comparison studies have been conducted to assess the performance of individual miRNA profiling platforms on a variety of sample types^19–23^. In 2014, a miRNA quality control (miRQC) study that evaluated 12 different technology platforms was published, which reported that the majority of platforms detected fewer than 90 miRNAs in human serum^19^. Since the miRQC study, newer platforms have emerged and reagent updates have been provided for several pre-existing platforms, but their relative performance in human biofluids is unclear^24–33^. Hence, an updated evaluation of miRNA quantitation platforms with a specific focus on human serum and plasma will be helpful. Here, we describe a systematic evaluation of four different qPCR platforms (MiRXES, Qiagen, Applied Biosystems, Exiqon), the NanoString nCounter platform, and the miRNA-Seq platform (Illumina TruSeq and Bioo Scientific NEXTflex) in measuring more than 600 miRNAs in human serum and plasma samples.

## Results

### Study design

**Figure 1:**
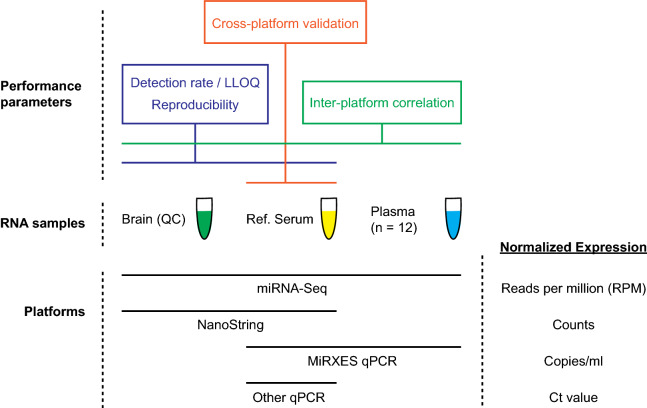
Overview of microRNA quantitation platforms, RNA samples, and different aspects of platform performance that were assessed in this study. Other qPCR platforms are Qiagen, ABI and Exiqon.

Our cross-platform evaluation relied on a set of reference samples (serum, plasma and brain) on which miRNA profiling was performed using six different platforms: (1) miRNA-Seq, (2) NanoString, (3) ABI Taqman qPCR. (4) Exiqon LNA qPCR, (5) Qiagen miScript qPCR and (6) MiRXES ID3EAL qPCR (Fig. 1). Each platform was assessed using performance parameters such as reproducibility, detection rate, and inter-platform correlation. The Reference Serum (Ref. Serum) is a large stock of pooled human serum that was purchased commercially. MiRNA profiling was performed on all six platforms using a similar input volume of Ref. Serum. The Human Brain Reference RNA sample has been characterized extensively by the MicroArray Quality Control (MAQC) project and was used in this study as a QC sample for the NanoString and miRNA-Seq platforms (hereafter referred to as ‘Brain QC sample’). On each platform, miRNA profiling was performed using the standard protocol from the manufacturer.

### miRNA profiling platforms

The four qPCR platforms evaluated in this study have slightly different methods for miRNA profiling (Table 1). For example, the ABI and MiRXES platforms rely on gene-specific primers for reverse transcription, but the Qiagen and Exiqon platforms perform universal A-tailing. The most comprehensive miRNA panels available from each manufacturer at the time of experiment were selected. Qiagen’s miRNome panel had the highest number of mature miRNAs (1066 miRNAs) whereas the MIRXES panel was the most focused (560 miRNAs) (Table 2). The number of overlapping miRNAs across the four qPCR panels is 438, significantly higher than the 196 overlapping miRNAs analyzed in the miRQC study^19^. Expression levels of miRNAs in the Qiagen, Exiqon and ABI panels were determined using Ct values and normalized by spike-in RNA or endogenous small RNA and/or calibrators accordingly to manufacturers’ recommendations.

**Table 1:** Overview of qPCR assays.

Workflow stage	Assay component	Qiagen miScript	Exiqon miRCURY	ABI Taqman	MiRXES ID3EAL
cDNA synthesis	Reverse transcription primer	Universal A-tailing	Universal A-tailing	miRNA-specific; universal stem-loop	miRNA-specific; modified stem loop
Amplification	Reverse PCR Primer	Universal primer	miRNA-specific LNA primer	Universal primer	miRNA-specific DNA primer
	Forward PCR Primer	miRNA-specific DNA primer	miRNA-specific LNA primer	miRNA-specific DNA primer	miRNA-specific DNA primer
Detection	Fluorescence	SYBR Green	SYBR Green	Taqman Probe	SYBR Green

**Table 2:** Platform comparison.

Technology	miRNA-Seq	nCounter	qPCR			
Platform	Illumina TruSeq	NanoString	Qiagen miScript	Exiqon miRCURY	ABI Taqman	MiRXES ID3EAL
Panel content (number of miRNAs)	Up to 2588^a^	800	1066	751	733	560
Turnaround time (days)^b^	4	2	7	5	5	7
Reagent cost per sample	+	+	++	++	++	++
Concordance correlation coefficient^c^	0.99	0.82	0.91	0.95	0.96	0.99
microRNAs detected above LLOQ	372^d^	84	NA	208	179	438

^a^Number of mature microRNAs in miRBase release 21.

^b^To obtain expression data for 12 samples using one instrument.

^c^Average of pairwise comparisons across a minimum of three independent runs.

^d^Based on sequencing depth of 20 million reads.

The NanoString assay involves ligating unique oligonucleotide tags onto target miRNAs, followed by detection of targets by hybridization to color-coded probes. Each target-probe pair is individually resolved and counted on the instrument. The nCounter Human v3 miRNA codeset contains probes for measuring 800 miRNAs (Table 2).

As the relative performance of miRNA-Seq library preparation kits from different manufacturers was unclear, we evaluated the performance of two different kits on our reference samples: the TruSeq Small RNA Library Prep Kit (Illumina) and NEXTflex Illumina Small RNA Sequencing Kit v2 (Bioo Scientific). Ultimately, we selected the TruSeq method for miRNA-Seq as our evaluation showed that the TruSeq kit is superior to the NEXTflex kit in overall yield and consistency of library preparation, miRNA detection rate and total hands-on time (detailed report in Supplementary Note 1). The miRNA-Seq assay relies on the presence of 5′-phosphate and 3′-hydroxyl groups on mature miRNAs to ligate specific RNA adapters onto each end of the target molecule. Reverse transcription is used to generate cDNA, which is then amplified by PCR to generate molecules that are compatible for sequencing on an Illumina sequencer. MiRNA expression levels are obtained by counting the number of sequence reads that align to regions of the human genome corresponding to mature miRNA sequences. We observed lower alignment rates in Ref. Serum and plasma samples (~ 9% and ~ 16% respectively) compared to the Brain QC sample (~ 38%), which may be due to a lower overall miRNA content in the biofluid samples (Table 3). In miRNA-Seq, the count data for each sample is normalized to sequencing depth and is represented as the number of reads per million mapped miRNA reads (RPM). In principle, miRNA-Seq can capture the complete range of miRNA species in a sample and has the potential to measure the largest number of miRNAs (Table 2). As the platform is sequence agnostic, miRNA-Seq can be used to identify and quantitate novel miRNAs, i.e., sequences that are likely to be miRNAs but are not annotated in miRBase.

**Table 3:** Summary statistics from miRNA-Seq.

	Ref. Serum (n = 3)^a^	Plasma (n = 12)^b^	Brain QC (n = 4)^a^
Sequence reads (A)	55,628,822	26,555,049	36,459,035
Mature microRNA reads (B)	4,969,597	4,330,330	13,985,954
Alignment rate (B/A)	8.9%	16.3%	38.4%
microRNAs with ≥ 5 reads	655	595	1035

^a^Average of inter-run replicates (number of replicates in parentheses).

^b^Average of plasma libraries from 12 patients.

### Reproducibility

On each platform, at least three independent runs were performed on Ref. Serum to obtain miRNA expression profiles. The concordance correlation coefficient (ccc) was calculated between runs to assess the reproducibility of each platform (Supplementary Fig. S1). We observed that the MiRXES qPCR and miRNA-Seq platforms had almost perfect concordance between runs (ccc = 0.99), the other three qPCR platforms had moderate inter-run concordance (ccc > 0.9), and the NanoString platform had poor inter-run concordance (ccc = 0.82) (Table 2). However, expression profiles obtained from the Brain QC sample had almost perfect inter-run concordance on both the miRNA-Seq and NanoString platforms (ccc = 0.99), suggesting that the lower reproducibility observed with NanoString may be restricted to sample types with lower miRNA content, such as serum (Supplementary Fig. S1). The miRNA-Seq method was also highly robust, as miRNA expression levels obtained from libraries with different input volume or PCR cycle number were highly concordant (Supplementary Table S1).

### Detection rate and lower limit of quantitation

**Figure 2:**
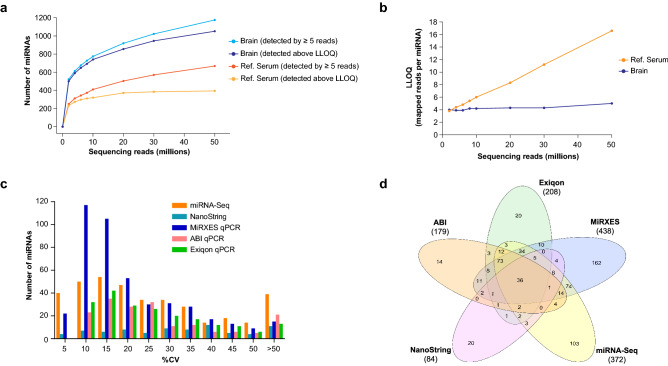
Detection rate, inter-platform overlap and LLOQ. (**a**) Detection rate of miRNAs as a function of sequencing depth for Brain QC and Ref. Serum miRNA-Seq libraries. (**b**) LLOQ of Brain QC and Ref. Serum miRNA-Seq libraries as a function of sequencing depth. LLOQ is expressed as the number of mapped reads per miRNA. (**c**) Coefficient of variation (%CV) observed in expression level for miRNAs detected above the LLOQ. (**d**) Overlap in miRNAs detected above LLOQ across platforms.

Detection rate is determined by a combination of platform content, sensitivity, and the platform-specific cutoff used for the limit of detection (LOD). Each platform’s lower limit of quantification (LLOQ) is the lowest number of miRNAs that can be quantitatively determined with a defined precision. In this study, LLOQ was determined using a cutoff of 50% coefficient of variation (CV) on all platforms. Using a LOD of five sequence reads, the number of unique miRNAs detected by miRNA-Seq in the Brain QC and Ref. Serum samples did not saturate even at a sequencing depth of 50 million reads (Fig. 2a). At this read depth, greater than 1150 and 650 miRNAs were detected above the LOD in the Brain QC and Ref. Serum samples, respectively. This suggests that miRNA-Seq can achieve very high sensitivity in biofluid samples by capturing a complex library of miRNA molecules, followed by deep sampling by next-generation sequencing. For miRNA-Seq, the LLOQ in Ref. Serum increases with higher sequencing depth, and was approximately eight reads at a sequencing depth of 20 million reads (Fig. 2b). In the Brain QC sample, the LLOQ was approximately four reads and was relatively stable at different sequencing depths (Fig. 2b). Our subsampling analysis suggests that miRNA-Seq libraries in serum should be sequenced to ~ 20 million reads as the number of miRNAs that can be reliably detected appears to reach saturation at this sequencing depth (Fig. 2a). However, as the number of additional miRNAs detected above LLOQ in Ref. Serum is relatively small when sequencing depth was increased from 10 to 20 million reads (300 vs 372 miRNAs), sequencing more samples at a lower depth of 10 million reads may be preferable in biomarker projects where budgets are limited. Conversely, the number of miRNAs that were detected above LLOQ in the Brain QC sample did not saturate even at a sequencing depth of 50 million reads (Fig. 2a). For tissue samples with more complex miRNA content, a trade-off will have to be made between increasing miRNA detection with sequencing depth and enhancing statistical power with more samples.

A more meaningful performance parameter is the number of miRNAs that can be reliably detected in a given sample. To that end, we calculated the LLOQ for each platform using expression data obtained from at least three independent runs (Online Methods, Supplementary Fig. S2). Overall, there were significant differences in the number of miRNAs detected above the LLOQ in Ref. Serum—NanoString only detected 84 miRNAs, miRNA-Seq detected 372 miRNAs, and the number of miRNAs detected by qPCR was highly variable between platforms (Table 2). Across all platforms, the MiRXES qPCR platform detected the highest number of miRNAs above the LLOQ. Notably, the LLOQ could not be calculated for the Qiagen qPCR platform as the variability in measurement of miRNA levels was particularly high even at the lower Ct range (Supplementary Fig. S2) and results from this platform were excluded from further analysis.

For miRNAs detected above the LLOQ, their expression levels were measured with highest precision by qPCR, compared to miRNA-Seq and NanoString (Fig. 2c).

### Inter-platform correlation

A total of 626 miRNAs were detected above the LLOQ in Ref. Serum by at least one platform (Fig. 2d). A significant proportion of miRNAs (51%) were only detected by one platform, and only 36 miRNAs (6%) were detected by all five platforms (Fig. 2d). Among the qPCR platforms, the largest inter-platform overlap with miRNA-Seq was observed with MiRXES (243 miRNAs), followed by Exiqon (167 miRNAs) and ABI (142 miRNAs) (Supplementary Table S2). The discrepancy in detection rate between the qPCR platforms could be partially explained by the higher overall sensitivity of the MiRXES ID3EAL assay—a significant number of miRNAs (246 miRNAs) were only detected by MiRXES but not Exiqon or ABI. However, a substantial proportion of the miRNAs detected by Exiqon (21%) and ABI (20%) were not detected above the LLOQ by MiRXES. Among these miRNAs, around 70% were missing from the MiRXES panel, indicating that a reason for the discrepancy is non-overlapping panel content across qPCR platforms. However, each qPCR platform may also be unique in their ability to reliably detect some miRNAs.

**Figure 3:**
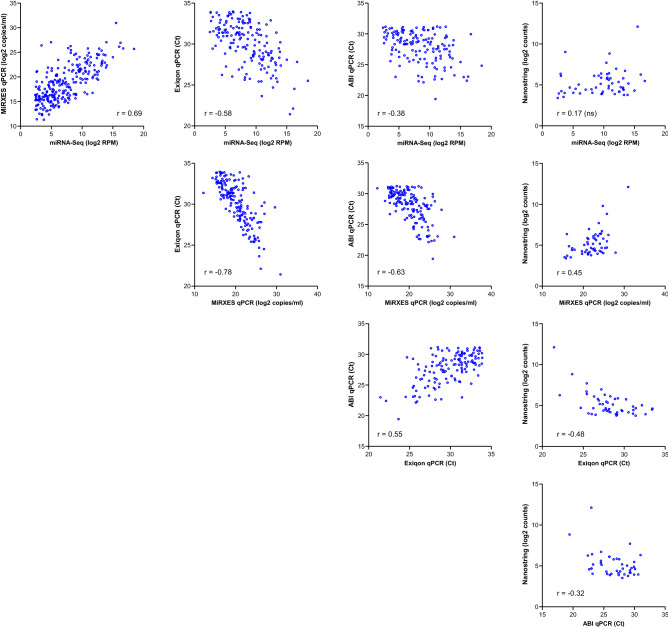
Inter-platform correlation. Pairwise inter-platform correlation in expression levels for miRNAs that were detected above the LLOQ in Ref. Serum. The miRNAs measured by qPCR do not include those in the “expanded” panel (see text). *r* Spearman’s correlation coefficient, *ns* not significant (p > 0.05).

Next, we evaluated inter-platform correlation in miRNA levels measured in Ref. Serum (Fig. 3). Overall, moderate to strong correlation was observed between the qPCR platforms and between qPCR and miRNA-Seq, except for ABI qPCR and miRNA-Seq where inter-platform correlation is weak (r = 0.38). The highest inter-platform correlation was observed between Exiqon qPCR and MiRXES qPCR (r = 0.78). Similar dynamic range in miRNA levels was detected by miRNA-Seq and qPCR (~ 10^6^). The correlation observed for NanoString with the other platforms were either not statistically significant (miRNA-Seq) or in the weak to moderate range (qPCR)—this could partly be explained by the lower dynamic range in miRNA detection for the NanoString platform (~ 10^3^). In contrast, substantial overlap and strong inter-platform correlation was observed between miRNA-Seq and NanoString in the Brain QC sample (r = 0.63); however, miRNAs detected by NanoString appear to be skewed towards lower expression levels, i.e., below 100 counts (Supplementary Fig. S3). These results suggest that lower detection sensitivity for the NanoString platform may be affecting its accuracy in measuring miRNA expression, particularly in samples with low miRNA content. Interestingly, strong correlation was observed between miRNA-Seq and MiRXES qPCR (r = 0.69); it was the second highest correlation observed between any two platforms in this study and this was comparable to that observed in twelve plasma samples on which miRNA profiling was also performed by both platforms (Supplementary Fig. S4).

Since miRNA-Seq and MiRXES qPCR had strong inter-platform correlation and higher assay precision and detection rate compared to the other platforms evaluated in this study, we decided to explore the potential of using miRNA-Seq as a discovery platform and MiRXES qPCR for validation. First, we evaluated how the MiRXES panel content for MiRXES qPCR affected inter-platform variation in miRNA detection. Among the miRNAs that were detected above the LLOQ by miRNA-Seq but not MiRXES qPCR, 83% (107 miRNAs) were absent from the MiRXES panel. Subsequently, we designed an “expanded” qPCR panel by developing new qPCR assays for 92 out of 107 miRNAs. When these 92 miRNAs were measured in Ref. Serum, 77 were detected above the LLOQ and their expression levels measured by qPCR had moderate correlation with miRNA-Seq (r = 0.45). Using the expanded MiRXES qPCR panel enabled the measurement of 320 miRNAs above LLOQ by both miRNA-Seq and qPCR in Ref. Serum (Supplementary Fig. S5) and 517 miRNAs by qPCR alone.

### Validation of novel serum miRNAs detected by miRNA-Seq

**Figure 4:**
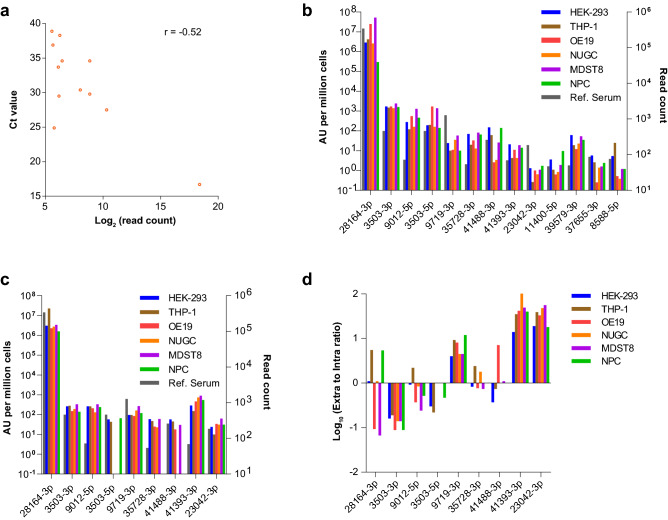
Validation of 14 novel miRNAs identified in Ref. Serum. (**a**) Correlation in expression levels of predicted mature miRNAs measured by qPCR and miRNA-Seq in Ref. Serum. Two miRNAs were not detected above the LOD (data points not shown). *r* Spearman’s correlation coefficient. (**b**) Intracellular expression levels of 13 novel miRNAs measured by qPCR across six human cell lines (left y-axis) and read count measured by miRNA-Seq in Ref. Serum (right y-axis). One miRNA was not detected above LOD (data points not shown). (**c**) Extracellular expression levels of nine novel miRNAs measured by qPCR across six human cell lines (left y-axis) and read count measured by miRNA-Seq in Ref. Serum (right y-axis). Five miRNAs were not detected above LOD (data points not shown). (**d**) Ratio of extracellular to intracellular expression.

Using miRNA-Seq as a discovery platform can also enable the identification of novel miRNAs that can be verified subsequently by qPCR. Novel miRNAs that were identified by miRNA-Seq in Ref. Serum were filtered for those that had a mirDeep score cutoff of ≥ 1 in technical replicates that were performed across three independent runs. The hairpin structure, mapped read distribution, and read counts observed in each predicted miRNA precursor suggested that they encoded true miRNAs (Supplementary Note 2). A total of 14 mature miRNA sequences that had average read counts per library ranging from 47 to 342,186 were selected for subsequent validation (Supplementary Table S3). The expression of these 14 miRNAs was measured by qPCR in Ref. Serum. Twelve miRNAs were detected above the LOD of the MiRXES platform (Ct < 40). We observed an inverse correlation between Ct values by qPCR and read counts by miRNA-Seq (Fig. 4a), although the correlation was not statistically significant at p = 0.05 (p = 0.08).

Next, we surveyed the intracellular and extracellular expression levels of these 14 predicted miRNAs by qPCR across six human cell lines of diverse origin—kidney (HEK-293), monocytic (THP-1), oesophageal (OE19), stomach (NUGC-3), colorectal (MDST8) and neural (NPC). Except for 31893-3p, which was also not detected by qPCR in Ref. Serum, intracellular expression for the remaining 13 miRNAs were detected at varying levels across the six cell lines (Fig. 4b). Notably, all 13 miRNAs were detected above the LOD in all cell lines. In contrast, extracellular expression was detected above the LOD for nine miRNAs in at least one cell line (Fig. 4c). Interestingly, only six miRNAs had detectable extracellular levels across all cell lines. For instance, 3503-5p was detected at relatively high levels (~ 27 Ct) in the cell culture media from HEK-293, THP-1 and NPC but was undetectable in OE19, MDST8 and NUGC-3, suggesting that this particular miRNA may not be secreted by cells of gastrointestinal origin. Further, we calculated the ratio of miRNA expression in the extracellular and intracellular fraction by normalizing for cell number and volume of culture media (Fig. 4d). This analysis revealed several interesting observations. Several miRNAs such as 41393-3p and 23042-3p were detected at substantially higher levels in the extracellular fraction across all cell lines (ratio > 10), suggesting that they were likely to be actively secreted by these cells. On the other hand, several miRNAs had ratios greater than or lesser than one in different cell lines. For instance, 28164-3p had a ratio of 5.5 and 0.1 in THP-1 and MDST8 cells respectively, which indicates that this miRNA is more likely to be secreted by the former. It should be noted that that intracellular RNA and extracellular RNA were extracted using different reagents, which may introduce bias in measuring absolute expression^34–36^.

## Discussion

Overall, our results show that miRNA profiling by qPCR, NanoString and miRNA-Seq differed significantly in performance, cost and speed (Table 2). Using a similar cutoff of 50% CV across all platforms, we found that MiRXES qPCR detected the highest number of miRNAs above the LLOQ in serum. This could be due to the use of a curated panel (optimized for human biofluids) and the high sensitivity and precision that is typically associated with qPCR technology. However, we also observed significant variability in performance in the other qPCR platforms evaluated in this study. Compared to MiRXES qPCR, miRNA-Seq detected slightly fewer miRNAs above the LLOQ in serum, but offers several advantages such as lower cost and faster turnaround time. Furthermore, the miRNA-Seq assay is target agnostic and will not require sample-specific optimization. The NanoString platform detected the lowest number of miRNAs above the LLOQ in serum. This could be due to several reasons: (a) in contrast to qPCR and miRNA-Seq, the NanoString assay is likely to have lower sensitivity as it does not involve target amplification, (b) the NanoString assay only allows a maximum RNA input volume of 3 µl, which is slightly lower than that of miRNA-Seq and qPCR. On the latter, we have found that using higher amounts of RNA input can increase the number of miRNAs detected above the LLOQ on the NanoString platform (data not shown). Therefore, it is likely that the performance of the NanoString platform on biofluid samples can be improved by increasing the overall RNA concentration of the sample, e.g., using vacuum concentration or a starting with higher volume of serum or plasma.

MiRNA biomarker development and successful translation into the clinic will require a robust assay that is capable of performing reliable measurement of miRNA abundance. Therefore, applying an LLOQ cutoff during biomarker discovery would be a reasonable approach to select candidates for subsequent follow up. To that end, we found that the number of miRNAs that can be reliably measured in serum reaches saturation at a sequencing depth of ~ 20 million reads. We suggest that this fit-for-purpose approach to validate the miRNA-Seq assay can maximize cost effectiveness by providing a rational basis for the amount of sequencing required per sample.

The platform-specific biases in miRNA detection that we observed in biofluids are not surprising, given that similar observations were made in other sample types. Nonetheless, we observed a high degree of overlap and strong correlation between miRNA-Seq and qPCR. Even though qPCR platforms are limited to the number of assays available, we showed that using miRNA-Seq to identify candidate miRNA biomarkers for subsequent validation by qPCR is a viable approach. The use of an expanded panel allowed us to perform qPCR-based measurements for the majority (86%) of miRNAs that were detected by miRNA-Seq, which resulted in the co-detection of 320 miRNAs on both platforms. To our knowledge, this is the largest number of miRNAs that can be reliably measured on two independent platforms in a single serum sample. Given the strengths and weaknesses of each platform, we propose that using miRNA-Seq for miRNA biomarker discovery and subsequent validation using qPCR represents a rational combination for miRNA biomarker development. However, our study did not assess the ability of each platform to detect differences between samples (e.g., healthy individuals and patients), so the potential of this approach will have to be evaluated in a subsequent study.

Another advantage of using miRNA-Seq for biomarker discovery is its ability to identify novel miRNAs. Of the 14 mature miRNAs that were selected for further analysis, most of them were also detected by qPCR in Ref. Serum. The existence of these predicted miRNAs in the serum suggests that they might be actively or selectively secreted^37^. Alternatively, these miRNAs may also be released into the circulation as a by-product of cell death^37^. By measuring the intracellular and extracellular expression levels of these predicted miRNAs across multiple cell lines, we provide experimental validation for many of these novel miRNAs and evidence for their active and/or tissue-specific secretion into the circulation.

## Methods

### Ethics statement

All patients provided written informed consent according to the Declaration of Helsinki, and the study was approved by the institutional review board of the National University Hospital (Singapore). All methods in this study were performed in accordance with relevant guidelines and regulations.

### RNA samples

Reference Serum is a human serum stock that was created by pooling serum samples from different individuals (Innovative Research, Inc.). RNA extraction was performed on 200 µl aliquots of Reference Serum using the miRNeasy Serum/Plasma Kit (Qiagen) by following the manufacturer’s recommendations, with the following modifications: (a) a set of three proprietary spike-in control RNAs (20 nt, MiRXES) with sequences distinct from any of the 2588 annotated mature human miRNAs (miRBase release 21) were spiked into the sample lysis buffer (QIAzol Lysis Reagent, Qiagen) at high, medium and low levels prior to sample RNA isolation; (b) due to the low abundance of total RNA in serum, bacteriophage MS2 RNA was added into the lysis buffer (1 µg per ml of QIAZol) to improve RNA isolation yield; (c) after mixing with chloroform, centrifugation was performed at 18,000×*g* for 15 min at room temperature; (d) the final elution step was performed with 15 µl of RNase-free water. RNA extracted from multiple aliquots of Reference Serum was pooled for cross-platform evaluation of microRNA profiling. FirstChoice Human Brain Reference RNA (Thermo Fisher Scientific) was used as a QC sample for miRNA-Seq and NanoString. Plasma samples were obtained from patients undergoing bariatric surgery at the National University Hospital (NUH). RNA extraction on plasma samples were performed using the same procedure described above.

### Cell lines

The following human cell lines were obtained commercially and cultured using the manufacturer’s recommendations—HEK-293 (ATCC), THP-1 (ATCC), OE19 (ECACC), NUGC-3 (JCRB), MDST8 (ECACC). Neural progenitor cells (NPC) were derived from hESCs (Es Cell International Pte Ltd) using a previously published protocol^38^. All cell lines were cultured in T75 flasks with 20 to 30 ml of culture media for up to 48 h. Cells were grown at sub-confluent density to minimize the release of intracellular miRNAs into the culture media due to cell death. To isolate extracellular RNA, 1 ml of cell culture media was transferred to a 1.5 ml tube and subjected to centrifugation at 16,000×*g* for 15 min to remove cells and cell debris. 200 µl of the supernatant was harvested for RNA extraction using the same procedure described above for serum/plasma. To isolate intracellular RNA, cells were harvested by trypsin treatment (for adherent cell lines), followed by centrifugation at 200×*g* for 5 min. The resulting pellet was resuspended in 1 ml of TRI Reagent (Sigma Aldrich) and RNA extraction was performed following manufacturer’s instructions. The Ct values obtained by qPCR were converted by applying a sample-specific scaling factor to normalize for the number of cells or amount of cell culture media that was measured. The normalized expression level for each miRNA was expressed as Arbitrary Units (AU) per million cells.

### MiRXES qPCR

Ten microliters of serum or plasma RNA was reverse transcribed according to manufacturer’s instructions (MiRXES) on a Veriti Thermal Cycler (Applied Biosystems). Template-specific RT primers for 560 miRNAs were divided into ten primer pools (50- to 60-plex per pool). Each primer pool was used for a multiplex RT reaction with 1 µl of RNA. A 6-log serial dilution of synthetic templates (10^7^ to 10^2^ copies) for each miRNA and a non-template control (nuclease-free water spiked with MS2) were reversed-transcribed with the isolated sample RNA. This enabled absolute quantification of sample miRNA expression copy numbers through intrapolation using standards curves generated with synthetic miRNA. Each cDNA sample was pre-amplified by 14 cycles of PCR using Augmentation Primer Pools (MiRXES) on the Veriti Thermal Cycler. Single-plex qPCR reactions were performed on the pre-amplified cDNA sample using miRNA-specific qPCR assays and Xtensa Sybr Green qPCR Master Mix according to manufacturer’s instructions (MiRXES). All qPCR reactions were performed with technical duplicates on the ViiA qPCR system (384-well configuration, Applied Biosystems). Raw threshold cycle (Ct) values were calculated using the ViiA 7 RUO software with automatic baseline setting and a threshold of 0.5. After quantification, miRNA levels (in copies per ml of serum or plasma) were determined by intra-polation of the sample miRNA Ct value to the standard curve of its synthetic miRNA template (Supplementary Table S4). Technical variations were normalized by a set of three proprietary spike-in control RNAs that were spiked at high, medium and low concentration prior to RNA isolation. For the 92 new miRNA assays that were added to the expanded panel, specific miRNA RT-qPCR assays were developed and miRNAs were measured using a similar qPCR workflow described above. RT primers for the 92 miRNAs were divided into two primer pools (50–60-plex per pool). Each primer pool was used for a multiplex RT reaction with 1 µl of RNA.

### ABI qPCR

Ten microliters of serum RNA was reversed transcribed using Megaplex RT Primers, Human Pool Set v3.0 (PN 4444745) with the Taqman MicroRNA Reverse Transcription Kit (PN 4366596) on the Veriti Thermal Cycler following the manufacturer’s protocol. The cDNA was pre-amplified using Megaplex PreAmp Primers (PN 4444748) and TaqMan PreAmp Master Mix (PN 4,391,128) on a Veriti Thermal Cycler. The pre-amplified cDNA was diluted 1:4 in TE then diluted 1:100 in 1× TaqMan Universal Master Mix II (PN 4440048) before loading on the matching TaqMan Array Human MicroRNA A + B Cards Set v3.0 (PN 4444913). The TaqMan Array Cards were sealed, briefly centrifuged, and run on a ViiA qPCR system using a TaqMan Array Block. Raw Ct values were calculated using the ViiA 7 RUO software with automatic baseline setting and a threshold of 0.5 (Supplementary Table S5).

### Exiqon qPCR

Ten microliters of serum RNA with UniSp6 RNA spike-in was reverse transcribed using the Universal cDNA Synthesis Kit II (PN 203301) on the Veriti Thermal Cycler. The cDNA was diluted 1:100 in ExiLENT SYBR Green MM (PN 203,421) before loading on the microRNA Ready-to-Use PCR, Human panel I + II, V4.M (PN 203615). The PCR plates were sealed, briefly centrifuged and run on ViiA qPCR system (384-well configuration). Raw Ct values were calculated using the ViiA 7 RUO software with automatic baseline setting and a threshold of 0.5. A normalization factor for each sample was calculated from the residual Ct of UniSp6 and the inter-plate calibrator UniSp3 in comparison to their respective averages over all samples. The normalization factor was then subtracted from the raw Ct for the normalized Ct for each miRNA in that sample (Supplementary Table S6).

### Qiagen qPCR

Ten microliters of serum RNA with miRTC was reversed transcribed using the miScript II RT Kit (PN 218161) on a Veriti Thermal Cycler. The cDNA was pre-amplified using the miScript PreAMP Primer Mix Human miRNome (miRBase V16) (PN 331251, MBHS-3216Z) and miScript PreAMP PCR Kit (PN 331451) on a Veriti Thermal Cycler. The pre-amplified cDNA was diluted 1:5 in nuclease free water, followed by 1:40 dilution in QuantiTect Sybr Green Mix from the miScript SYBR Green PCR Kit (PN 218075) before loading on the matching miScript miRNA PCR Array Human miRNome (PN 331222, MIHS-3216Z). The PCR plates were sealed, briefly centrifuged and run on a ViiA qPCR system (384-well configuration). Raw Ct values were calculated using the ViiA 7 RUO software with automatic baseline setting and a threshold of 0.5. A normalization factor for each sample was calculated from the residuals of the Ct of miRTC and the PPC in comparison to their respective averages over all samples. The normalization factor was then subtracted from the raw Ct for the normalized Ct for each miRNA in that sample (Supplementary Table S7).

### NanoString

Three microliters of serum RNA and 100 ng of brain RNA were processed according to the manufacturer’s recommendations for the nCounter Human v3 miRNA Expression Assay Kit (NanoString; CSO-MIR3-12). Each sample was scanned for 555 FOV on the nCounter Digital Analyzer. Raw count data from independent runs of Reference Serum were processed together in nSolver (v2.0; NanoString). For each gene, count data was processed as follows: (a) background subtraction using the geometric mean of the negative controls, (b) normalization to the geometric mean of the positive controls, and (c) normalization to the geometric mean of the top 100 most highly expressed microRNAs.

### miRNA-Seq

miRNA-Seq libraries were prepared using the TruSeq Small RNA Library Prep Kit (Illumina; RS-200-0012) or the NEXTflex Illumina Small RNA Sequencing Kit v2 (Bioo Scientific; NOVA-5132-03). Brain QC libraries were prepared using 1 µg of FirstChoice Human Brain Reference RNA. For the TruSeq method, library preparation was performed using 5 µl of RNA following the manufacturer’s recommendations (Rev.F; February 2014). 11 cycles of PCR were performed for the Brain QC samples, 15 cycles of PCR were performed for the Reference Serum samples, and 18 cycles of PCR were performed for the plasma samples. For the NEXTflex method, library preparation was performed using 4 µl of total RNA following the manufacturer’s recommendations (V14.11). 15 cycles of PCR were performed for the Brain QC samples and Reference Serum samples.

For both methods, gel purification of the library construct was performed by ethanol precipitation. Purified libraries were verified on a High Sensitivity D1000 ScreenTape (Agilent Technologies) and quantified by real-time PCR using a Library Quantification Kit (KAPA Biosystems). Pooled libraries from each run were loaded at 1.8 pM for 1 × 40 bp sequencing on a NextSeq 500 using a High Output Kit (Illumina.). Sequence data in fastq format was obtained using bcl2fastq (v2.16).

The CAP-miRSeq pipeline^39^ was used to process and analyze miRNA-Seq data. In brief, reads were trimmed for low quality bases and Illumina adapter sequences using cutadapt^40^. For data generated using the NEXTflex method, additional trimming was performed to remove the randomized adapter sequences at the ligation junctions. Trimmed reads were aligned to the hg38 reference genome assembly (http://hgdownload.cse.ucsc.edu/goldenPath/hg38/bigZips/hg38.fa.gz) using the miRDeep2 package^41^. Expression levels for known microRNAs were quantified by counting reads mapping to microRNA coordinates defined in miRBase release 21 (ftp://mirbase.org/pub/mirbase/21) and novel microRNAs were also predicted by miRDeep2. Based on structure and read signature for each potential miRNA precursor, the mirDeep2 core algorithm assigns a score for each potential miRNA precursor that reflects the likelihood of it being a genuine miRNA. The CAP-miRSeq pipeline was run using default parameters. Within each sample, expression levels for mature microRNAs were normalized to **r**eads per million mapped reads (RPM).

### Statistical analyses

The lower limit of quantification (LLOQ) was determined using at least three independent runs on each platform. The microRNA expression data was fitted by LOESS (locally estimated scatterplot smoothing), to estimate the trend between mean expression (e.g., normalized counts or Ct values) and variability (i.e., coefficient of variation). LLOQ was determined using a cutoff of 50% CV on all platforms (Supplementary File 1). Lin’s concordance correlation coefficient^42^ was calculated using the epi.ccc function in the epiR package (https://cran.r-project.org/web/packages/epiR/index.html).

## Supplementary Information

Supplementary Information 1.

Supplementary Information 2.

Supplementary Information 3.

Supplementary Information 4.

Supplementary Information 5.

Supplementary Information 6.

## Data Availability

Raw data have been deposited in the NCBI Sequence Read Archive under study accession number SRP090487.
