# Supplementary material for: Systematic evaluation of multiple qPCR platforms, NanoString and miRNA-Seq for microRNA biomarker discovery in human biofluids
Source: Sci Rep. 2021 Feb 24;11:4435. doi: 10.1038/s41598-021-83365-z (PMC7904811; doi:10.1038/s41598-021-83365-z)
Supplement: Supplementary file 1 — Supplementary Information 1. [file 41598_2021_83365_MOESM1_ESM.pdf]

Systematic evaluation of multiple qPCR platforms, NanoString and miRNA-Seq for microRNA biomarker discovery in human biofluids

Lewis Z. Hong[1*], Lihan Zhou[2], Ruiyang Zou[2], Chin Meng Khoo[3], Adeline Lai San Chew[1], Chih-Liang Chin[1], Shian-Jiun Shih[1]

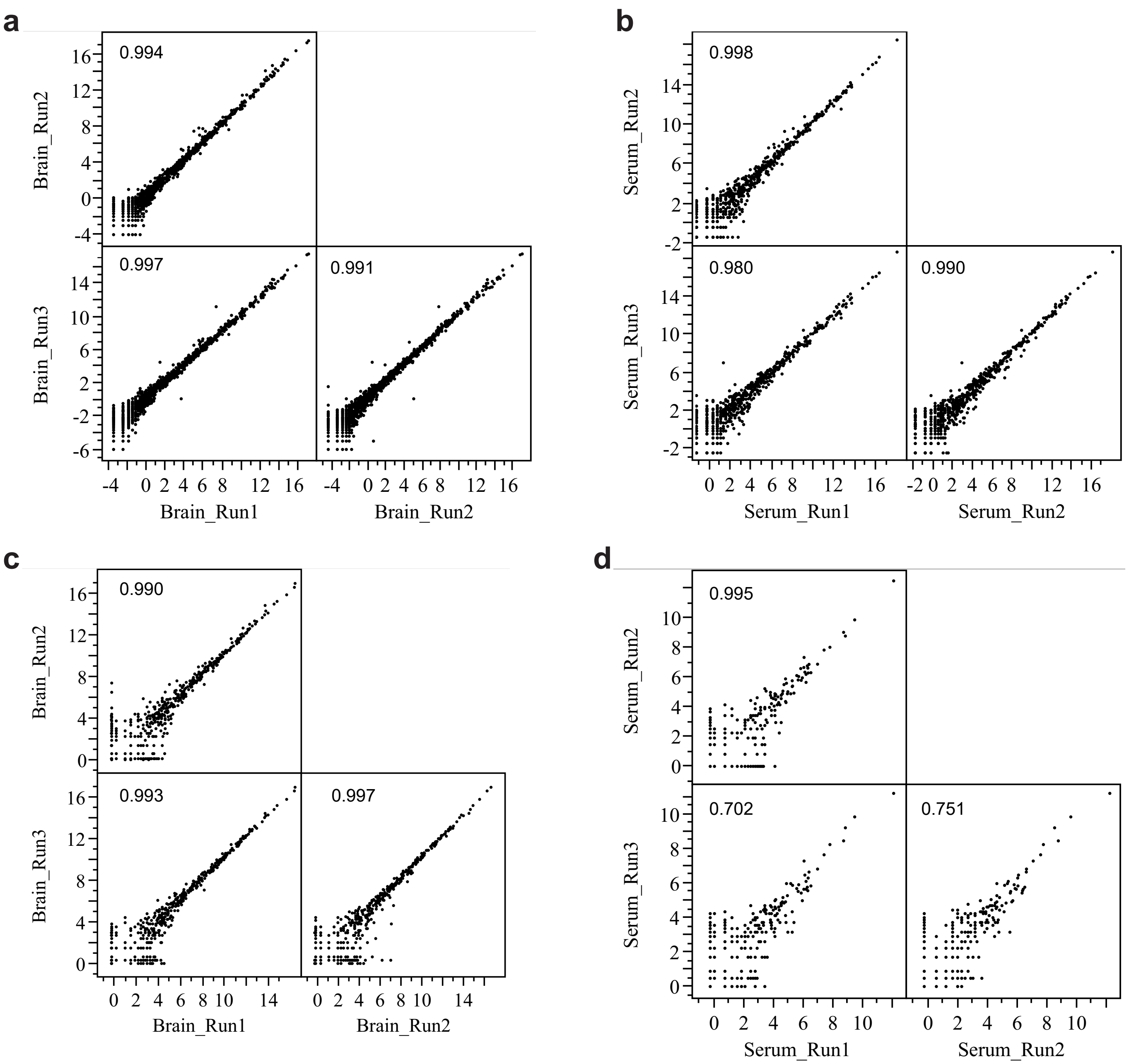

Supplementary Figure 1. Comparison of microRNA expression for inter-run replicates.
Pairwise scatterplots of microRNA expression profiles for Brain QC and Ref. Serum samples measured by (**a and b**) miRNA-Seq and (**c and d**) NanoString. Gene expression for each micoRNA is represented by RPM for miRNA-Seq libraries and normalized counts for NanoString. Concordance correlation coefficient for each pairwise comparison is shown. Axes are plotted in log_2 scale.

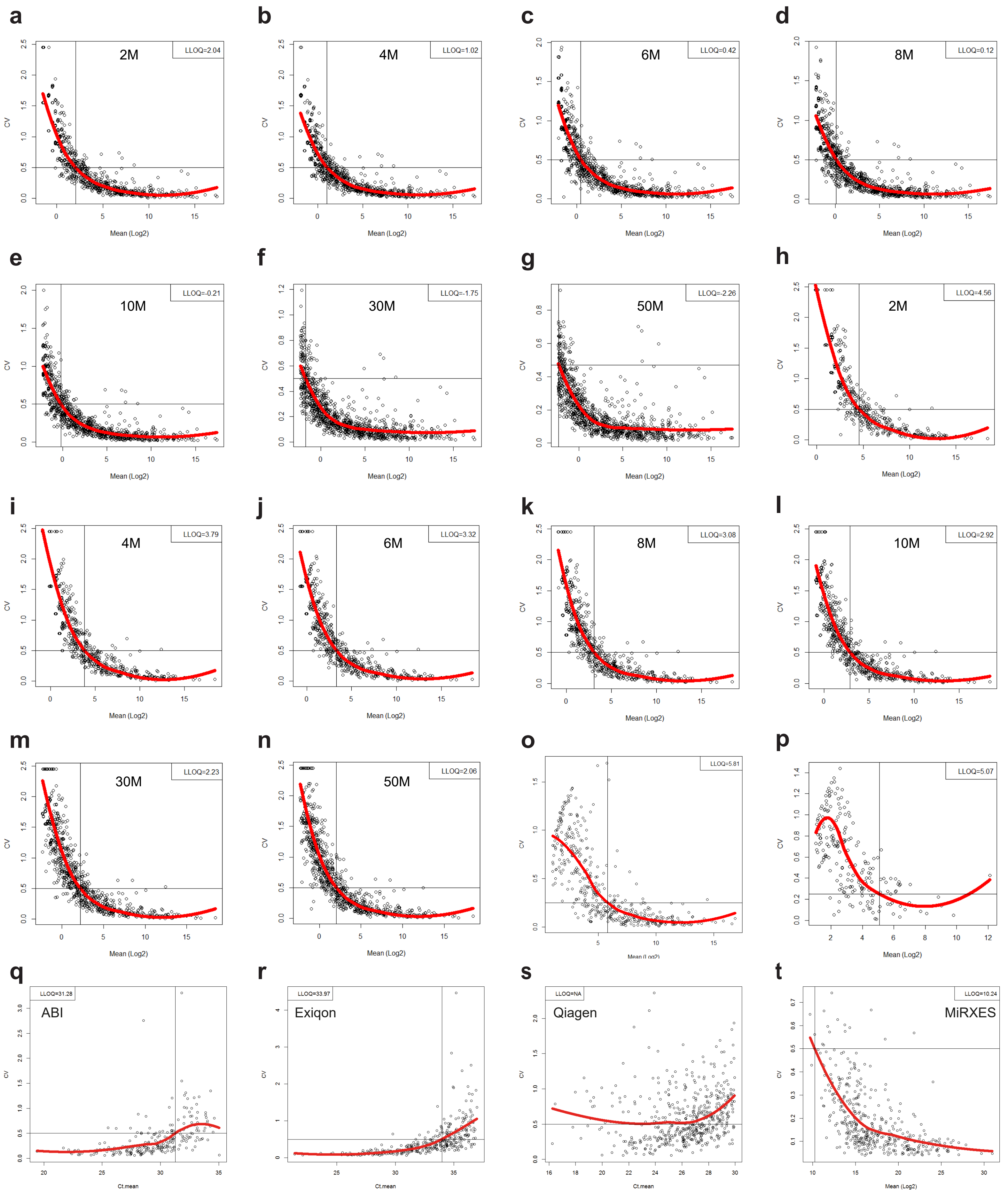

Supplementary Figure 2. LLOQ for miRNA-Seq, NanoString and qPCR.
The LLOQ for Brain (**a-g and o**) and Ref. Serum (**h-n and p-t**) samples on miRNA-Seq (**a-n**), NanoString (**o and p**) and qPCR (**q-t**) were determined by fitting the miRNA expression data by LOESS (locally estimated scatterplot smoothing) to estimate the trend between mean expression (across inter-run replicates) and variability (i.e., coefficient of variation). LLOQ was determined using a cutoff of 50% CV for all platforms. The sequencing depth per library is shown in each plot (e.g., 2M = 2 million reads).

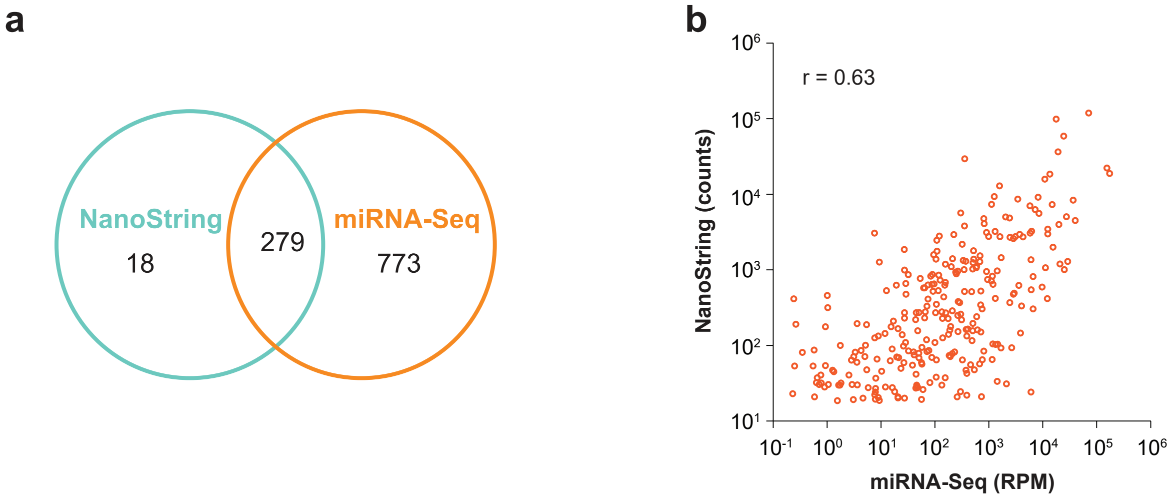

Supplementary Figure 3. Inter-platform overlap and correlation in Brain QC sample.
(a) Overlap in miRNAs detected above LLOQ in Nanostring and miRNA-Seq. (b) Correlation in expression levels between NanoString and miRNA-Seq for 279 miRNAs that were detected above the LLOQ. r: Spearman's correlation coefficient

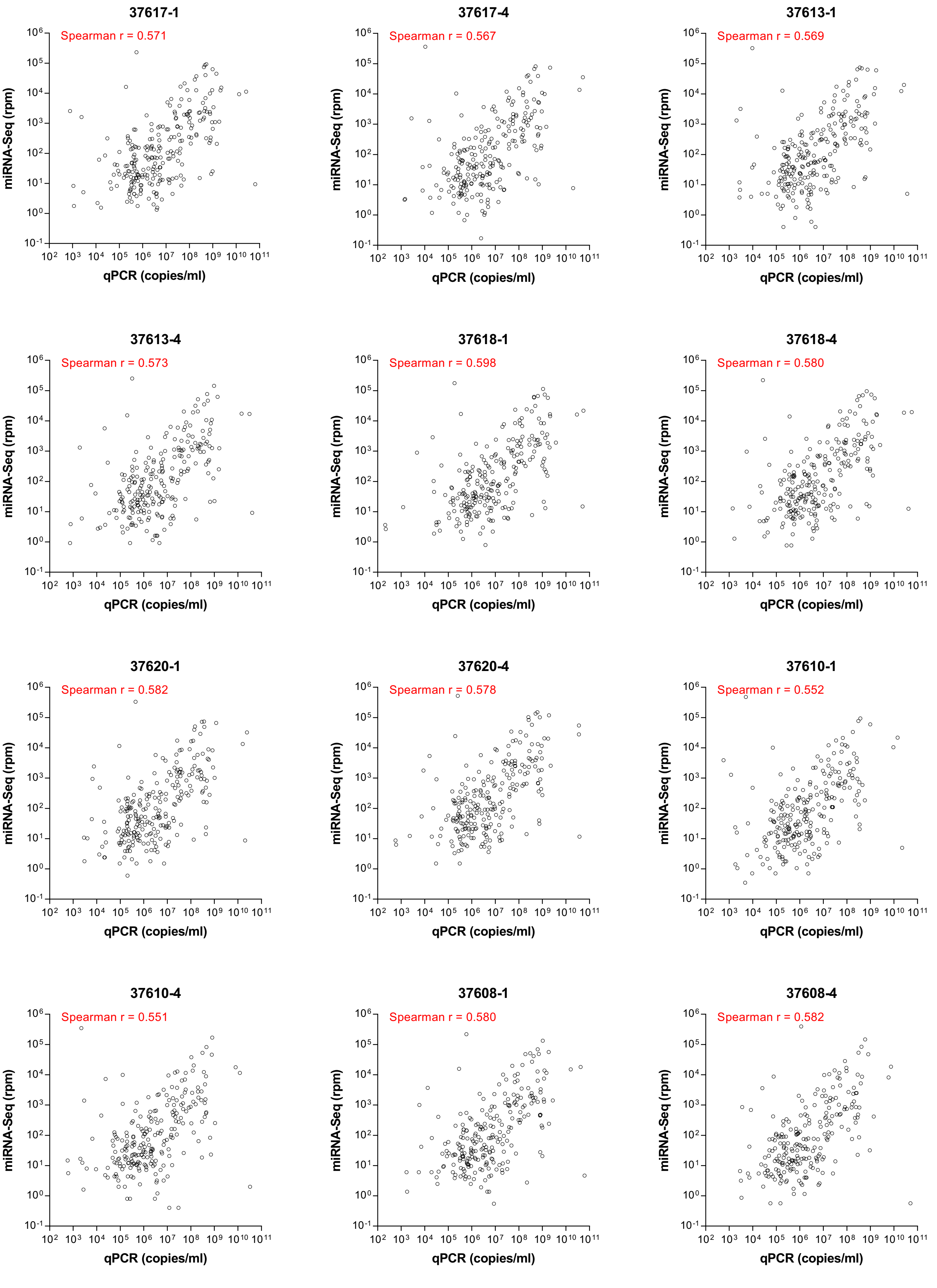

Supplementary Figure 4. Correlation of miRNA-Seq and MiRXES qPCR expression profiles in human plasma. Inter-platform correlation in miRNA expression in 12 human plasma samples. Only miRNAs that are present at ≥10 reads per sample by miRNA-Seq or ≥1000 copies/ml by qPCR are compared between the platforms.

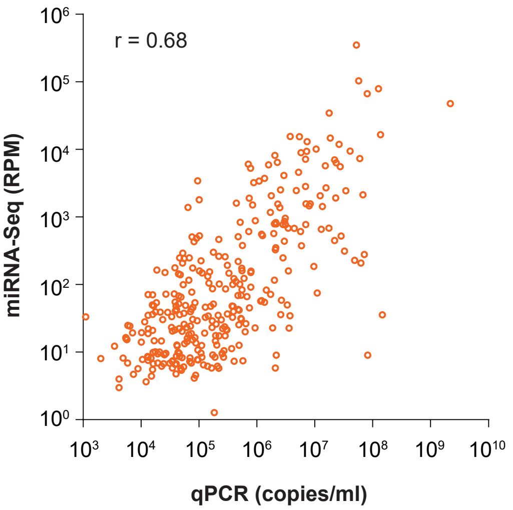

Supplementary Figure 5. Correlation of miRNA-Seq and MiRXES qPCR expression in Ref. Serum.
Correlation in miRNA expression levels in Ref. Serum. Using an expanded panel of qPCR assays that included new assays for miRNAs that were intially detected by miRNA-Seq, a total of 320 miRNAs were measured above the LLOQ by miRNA-Seq and qPCR. r: Spearman's correlation coefficient.
