# Supplementary material for: Systematic evaluation of multiple qPCR platforms, NanoString and miRNA-Seq for microRNA biomarker discovery in human biofluids
Source: Sci Rep. 2021 Feb 24;11:4435. doi: 10.1038/s41598-021-83365-z (PMC7904811; doi:10.1038/s41598-021-83365-z)
Supplement: Supplementary file 3 — Supplementary Information 3. [file 41598_2021_83365_MOESM3_ESM.pdf]

Supplementary Note 1

Systematic evaluation of multiple qPCR platforms, NanoString and miRNA-Seq for microRNA biomarker discovery in human biofluids

This Supplementary Note describes the evaluation of the TruSeq Small RNA Library Prep Kit (Illumina) and the NEXTflex Illumina Small RNA Sequencing Kit v2 (Bioo Scientific). Both methods were compared in terms of mapping rate, reproducibility, detection rate, and correlation in expression levels.

Overview

The protocols for the TruSeq and NEXTflex kits follow a similar procedure. In brief, both protocols rely on the presence of 5'-phosphate and 3'-hydroxyl groups on mature microRNAs (and other small RNAs) to ligate RNA adapters onto each end of the target molecule. Reverse transcription is used to generate cDNA, which is then PCR amplified using primers that contain index sequences for multiplexing. PCR products are pooled for gel purification to isolate libraries with the desired insert size for microRNAs.

The NEXTflex kit uses randomized adapters, i.e., each adapter contains four degenerate bases proximal to the ligation site, which is supposed to reduce ligation bias and more accurately represent the relative levels of microRNAs in a sample. While both kits have similar turnaround time, the NEXTflex kit has slightly longer hands-on time (~0.5 h) due to the inclusion of post-reaction cleanup steps using AMPure XP beads.

Samples

Reference Serum RNA and FirstChoice Human Brain Reference RNA (Thermo Fisher Scientific).

Summary statistics

Table 1 | Summary statistics from TruSeq and NEXTflex kits

	**Ref. Serum (n = 3)[1]**	**Brain (n = 3)[1]**	**Ref. Serum (n = 2)[1]**	**Brain (n = 3)[1]**
Kit	TruSeq	TruSeq	NEXTflex	NEXTflex
PCR cycles	15	11	18	15
Library concentration (nM)	23.8	32.9	1.5	16.5
Sequence reads (A)	55,628,822	36,459,035	17,330,136	51,056,009
Mature microRNA reads (B)	4,969,597	13,985,954	1,389,043	29,680,002
Alignment rate (B/A)	8.9%	38.4%	8.0%	58.1%
microRNAs with ⩾ 5 reads	655	1035	406	1039

[1] Average of inter-run replicates (number of replicates in parentheses).

From the same amount of input RNA, the library yield obtained using the TruSeq kit was higher (Table 1) and more consistent between replicates (data not shown). For the NEXTflex kit, a higher number of PCR cycles had to be performed on Ref. Serum in order to obtain libraries of sufficient yield for sequencing (Table 1). Overall, the alignment rate was higher for the TruSeq method using Ref. Serum RNA as input, but lower when the input was Brain RNA (Table 1).

Reproducibility

Figure 1 | Comparison of microRNA expression levels in inter-run replicates.

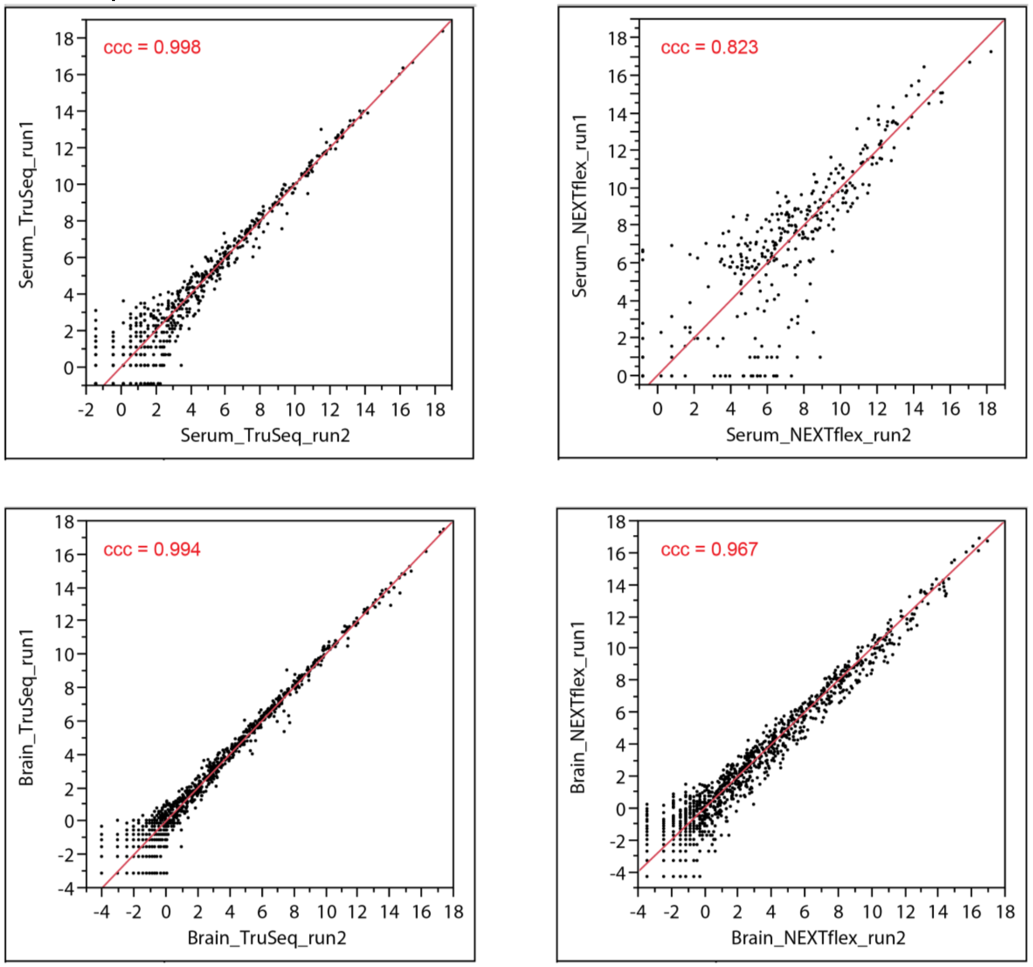

From the same aliquot of RNA from Ref. Serum and Brain, independent libraries—hereafter referred to as "inter-run replicates"—were generated using the TruSeq and NEXTflex kits. Comparisons of microRNA expression levels observed in inter-run replicates are shown in Figure 1. For each library, micoRNA expression levels were normalized to RPM and plotted on a $\log_2$ scale. Using the TruSeq kit, near perfect concordance was observed for Ref. Serum and Brain samples (concordance correlation coefficient, or ccc > 0.99). Using the NEXTflex kit, substantial concordance was observed for Brain (ccc > 0.95), but the concordance observed for Ref. Serum was poor (ccc < 0.85). These results indicate that microRNA expression levels measured using the TruSeq kit from serum were more reproducible compared to the NEXTflex kit.

Detection rate

Figure 2 | Detection rate in Brain and Ref. Serum

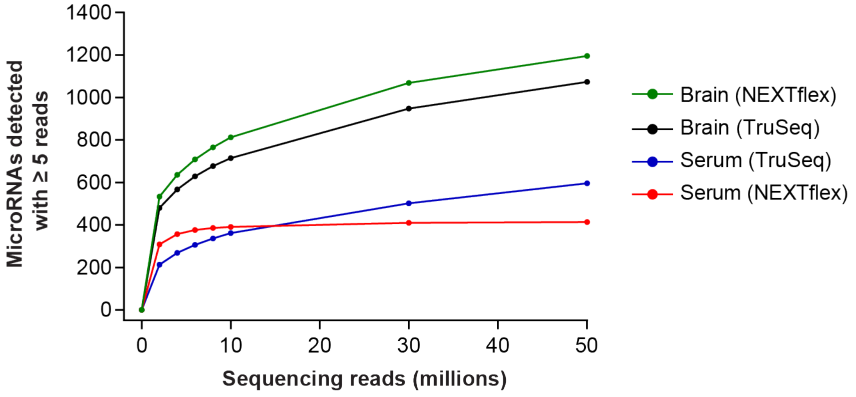

To assess the detection rate across libraries, each inter-run replicate was sequenced to a depth of at least 50 million reads. Subsampling analysis was performed to determine the number of microRNAs that were detected at different sequencing depth (Figure 2).

Libraries that were generated from Brain using the TruSeq and NEXTflex kits detected increasing numbers of unique microRNAs up to 50 million reads, indicating that libraries generated using either method were of high complexity. At various sequencing depths, the NEXTflex libraries detected more microRNAs compared to the TruSeq libraries. This is likely due to the higher alignment rate in the NEXTflex libraries (Table 1).

Libraries that were generated from Serum using the NEXTflex kit detected more unique microRNAs compared to the TruSeq libraries, but only up to ~10 million reads. Beyond 10 million reads, the NEXTflex libraries appear to be saturated, suggesting that NEXTflex libraries generated from serum have lower complexity compared to TruSeq libraries.

Overlap and correlation

Figure 3 | Overlap in microRNAs detected at ≥ 1 RPM

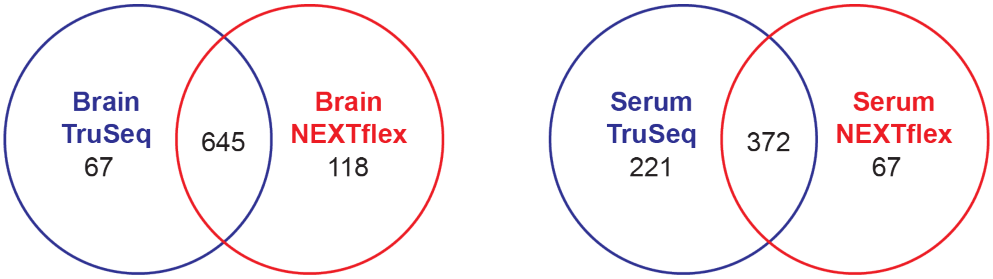

Figure 4 | Correlation in microRNA levels measured by each kit

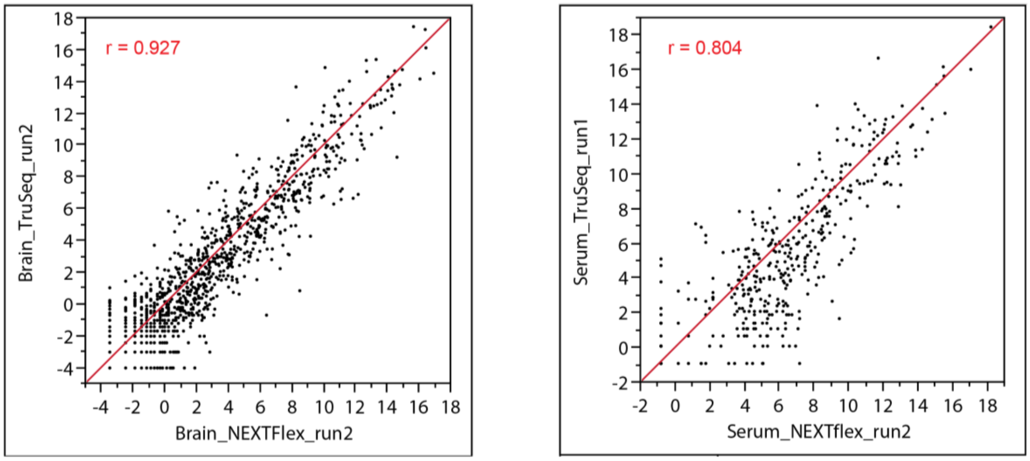

In Brain, 645 microRNAs were detected by both kits at above 1 RPM (Figure 3) and expression levels measured by both kits were strongly correlated (Pearson r = 0.927) (Figure 4). In Serum, only 372 microRNAs were detected by both kits at above 1 RPM (Figure 3) and the expression levels were more poorly correlated (r = 0.804) (Figure 4). Furthermore, a larger proportion of serum microRNAs were not detected by the NEXTflex kit, consistent with our observation that TruSeq libraries generated from serum were of higher complexity (Figure 2).

Summary

The TruSeq Small RNA Library Prep kit has several advantages over the NEXTflex Illumina Small RNA Sequencing kit v2. Compared to the NEXTflex kit, the TruSeq kit has slightly shorter hands-on time and generates higher yield libraries with higher reproducibility. In serum samples, TruSeq libraries have a higher detection rate beyond 10 million reads, suggesting that these libraries have higher complexity and the TruSeq kit may be more suitable for low input samples, i.e., samples with low microRNA content. Previously published studies had also observed a poorer performance of the NEXTflex method with low input samples [1,2]. However, it should be noted that our study involved the use of an older version of the NEXTflex kit. The NEXTflex method was designed to reduce bias in library preparation during the adapter ligation step, by increasing adapter sequence diversity [3]. The effect of sequence bias was not evaluated in this study due to the large differences in library complexity in Ref. Serum between the two methods; nonetheless it should be noted that reduced bias of NEXTflex vs TruSeq libraries had been observed in previous studies [4,5].

References

1 Wong, R. K. Y., MacMahon, M., Woodside, J. V. & Simpson, D. A. A comparison of RNA extraction and sequencing protocols for detection of small RNAs in plasma. *BMC Genomics* **20**, 446, doi:10.1186/s12864-019-5826-7 (2019).

2 Yeri, A. *et al.* Evaluation of commercially available small RNASeq library preparation kits using low input RNA. *BMC Genomics* **19**, 331, doi:10.1186/s12864-018-4726-6 (2018).

3 Baran-Gale, J. *et al.* Addressing Bias in Small RNA Library Preparation for Sequencing: A New Protocol Recovers MicroRNAs that Evade Capture by Current Methods. *Front Genet* **6**, 352, doi:10.3389/fgene.2015.00352 (2015).

4 Heinicke, F. *et al.* An extension to: Systematic assessment of commercially available low-input miRNA library preparation kits. *RNA Biol* **17**, 1284-1292, doi:10.1080/15476286.2020.1761081 (2020).

5 Srinivasan, S., Duval, M. X., Kaimal, V., Cuff, C. & Clarke, S. H. Assessment of methods for serum extracellular vesicle small RNA sequencing to support biomarker development. *J Extracell Vesicles* **8**, 1684425, doi:10.1080/20013078.2019.1684425 (2019).
