# Supplementary material for: Systematic evaluation of multiple qPCR platforms, NanoString and miRNA-Seq for microRNA biomarker discovery in human biofluids
Source: Sci Rep. 2021 Feb 24;11:4435. doi: 10.1038/s41598-021-83365-z (PMC7904811; doi:10.1038/s41598-021-83365-z)
Supplement: Supplementary file 4 — Supplementary Information 4. [file 41598_2021_83365_MOESM4_ESM.pdf]

Systematic evaluation of multiple qPCR platforms, NanoString and miRNA-Seq for microRNA biomarker discovery in human biofluids

Lewis Z. Hong[1*], Lihan Zhou[2], Ruiyang Zou[2], Chin Meng Khoo[3], Adeline Lai San Chew[1], Chih-Liang Chin[1], Shian-Jiun Shih[1]

Supplementary Note 2

This Supplementary Note provides the hairpin structure of thirteen predicted novel microRNA precursors that were reproducibly detected in Reference Serum by mirDeep2 across eight miRNA-Seq libraries (technical replicates) and were subsequently evaluated by qPCR. Each hairpin structure is represented here by the mapped read distribution observed in one of the replicate miRNA-Seq libraries.

Provisional ID	:	chr1_3503
Score total	:	141.4
Score for star read(s)	:	3.9
Score for read counts	:	141.9
Score for mfe	:	-2
Score for randfold	:	-2.2
Score for cons. seed	:	
Total read count	:	290
Mature read count	:	213
Loop read count	:	0
Star read count	:	77

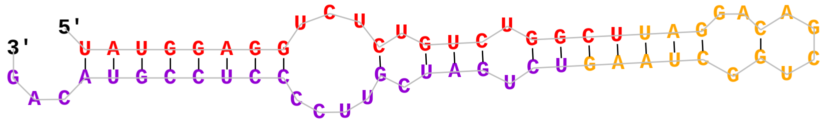

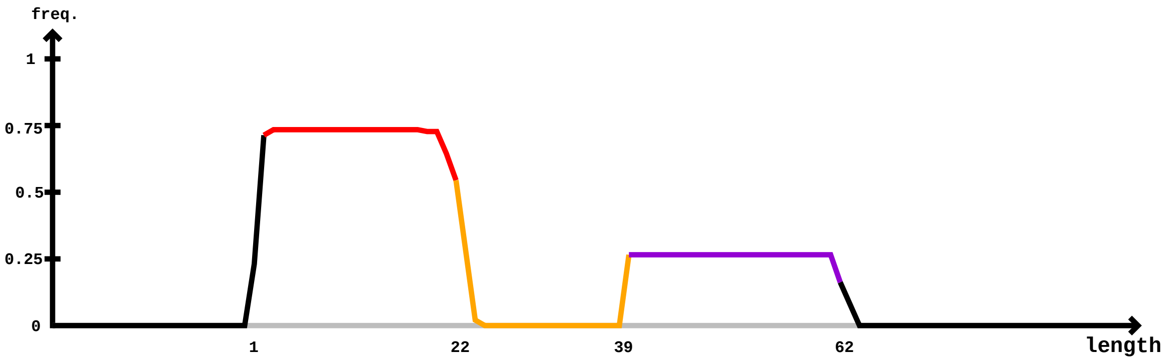

Mature **Star**

```
5'- gcagcgauccuacauaaauauauggaggucucugucuggcuuaggacagcuggcuaagucugaucguuccccuccguacagccuuuaaaacugccacucuaaauacuuggu   -3'    obs
    gcagcgauccuacauaaauauauggaggucucugucuggcuuaggacagcuggcuaagucugaucguuccccuccguacagccuuuaaaacugccacucuaaauacuuggu          exp
    ((((.(......).......((((((((...(.(((.(((((((..(....).))))))).))).)....))))))))...........)))).................   reads   mm   sample
    ...................auauggaggucucugucu..........................................................................   1       0    seq
    ...................auauggaggucucAgucu..........................................................................   1       1    seq
    ...................auauggaggucucugucuggc.......................................................................   14      0    seq
    ...................Guauggaggucucugucuggcu......................................................................   1       1    seq
    ...................auauggaggucucugucuggcA......................................................................   4       1    seq
    ...................auauggaggucucugucuggcu......................................................................   46      0    seq
    ....................uauggaggucucugucugg........................................................................   24      0    seq
    ....................uauggaggucucugucuggc.......................................................................   15      0    seq
    ....................uauggaggucucugucAggcu......................................................................   1       1    seq
    ....................uauggaggucucugucuggcA......................................................................   1       1    seq
    ....................uauggaggucucugucuggcu......................................................................   66      0    seq
    ....................uauggaggucucugucuggcuu.....................................................................   26      0    seq
    ....................Aauggaggucucugucuggcuu.....................................................................   1       1    seq
    ....................uauggaggucucugucuggcuuU....................................................................   6       1    seq
    .....................auggaggucucugucuggcu......................................................................   1       0    seq
    .....................auggaggucucugucuggcuu.....................................................................   5       0    seq
    ..........................................................ucugaucguuccccuccAuaca...............................   30      1    seq
    ..........................................................ucugaucguuccccuccAuacag..............................   41      1    seq
    ..........................................................ucugaucguuccccuccAuacagc.............................   6       1    seq
```

Provisional ID	:	chr3_8588
Score total	:	2.5
Score for star read(s)	:	-1.3
Score for read counts	:	0
Score for mfe	:	2.2
Score for randfold	:	1.6
Score for cons. seed	:	
Total read count	:	55
Mature read count	:	55
Loop read count	:	0
Star read count	:	0

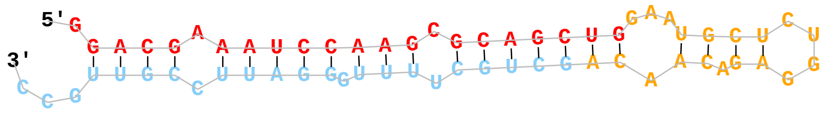

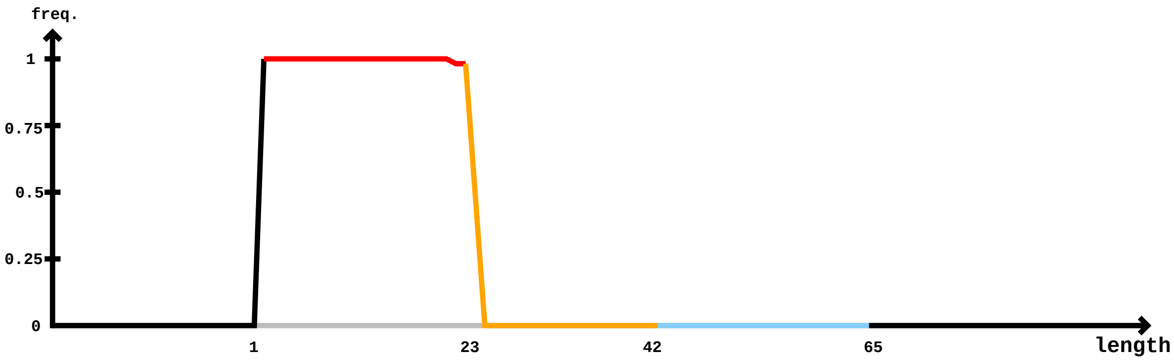

Mature Star

```
5'- ugcucaggcaggagagcaggggacgaaauccaagcgcagcuggaaugcucuggagacaacagcugcuuuugggauuccguugcccgcuguccagccguuggcggggggcggg   -3'    exp
    .((((.....(((.(((.((.((((.((((((((.(((((((...((((....)).)).)))))))).))).))))).)))).)).))).)))..(((....))).))))...    reads    mm    sample
    ....................ggacgaaauccaagcgcagc........................................................................    1        0     seq
    ....................ggacgaaauccaagcgcagcug......................................................................    42       0     seq
    ....................ggGcgaaauccaagcgcagcug......................................................................    1        1     seq
    ....................ggacgaaauccaagcgcagcugU.....................................................................    11       1     seq
```

Provisional ID	:	chr3_9012
Score total	:	1.6
Score for star read(s)	:	-1.3
Score for read counts	:	0
Score for mfe	:	1.3
Score for randfold	:	1.6
Score for cons. seed	:	
Total read count	:	91
Mature read count	:	91
Loop read count	:	0
Star read count	:	0

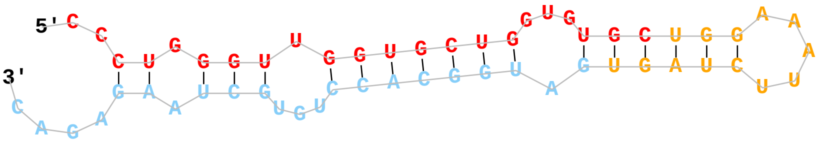

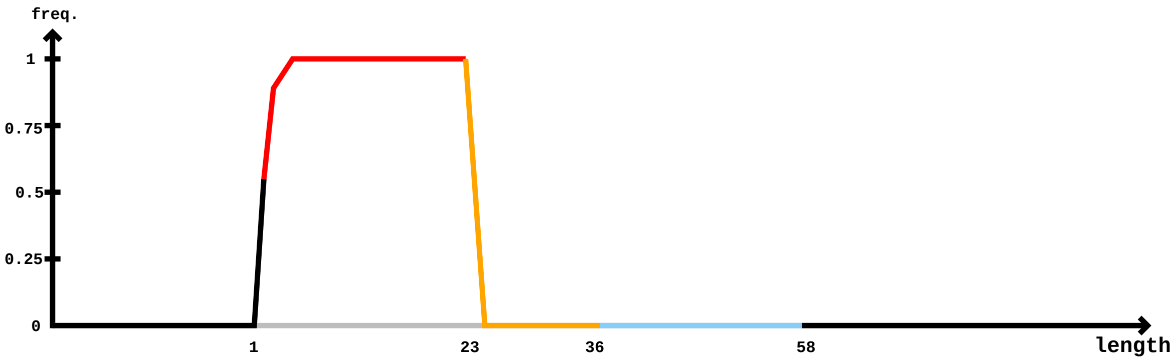

Mature **Star**

```
5'- cccuuugaugagaacauggucccugggguuggugcuggugugcuggaaauucuagugauggcaccugugcuaagagaccuuugaucuuaagagaaaaaaagguagaggagaga   -3'    exp
    .((((((.(((((.((.((((.((.(((.(((((((...((((((.....)))))).)))))))...))).)).)))).)).)))))............)))))).....    reads     mm      sample
    ....................cccugggguuggugcuggugugc......................................................................    50        0       seq
    .....................ccugggguuggugcuggugugc......................................................................    31        0       seq
    ......................cuggguuggugcuggugugc......................................................................    5         0       seq
    .......................uggguuggugcuggugugc......................................................................    5         0       seq
```

Provisional ID	:	chr3_9719
Score total	:	2.4
Score for star read(s)	:	-1.3
Score for read counts	:	0
Score for mfe	:	2.1
Score for randfold	:	1.6
Score for cons. seed	:	
Total read count	:	894
Mature read count	:	894
Loop read count	:	0
Star read count	:	0

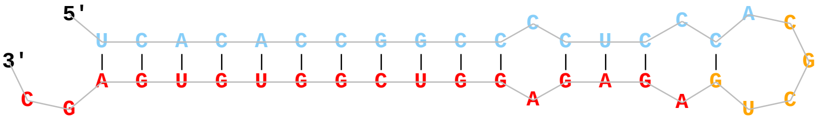

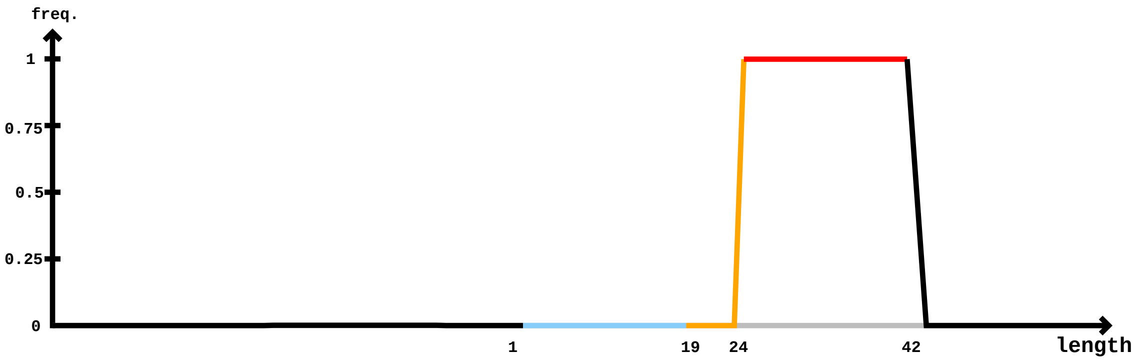

Star Mature

```
5'- acaccggccccucccacgcggacagaggucagcgugagccccuugccucacaccggccccucccacgcugagagaggucggugugagcccuugccucacaccggcccc   -3'   exp
    .....((((..(((.....)))..(((((..((....)).......((((((((((((.(((.(.....).))).))))))))))))).....))))).....))))..   reads   mm   sample
    .....................aGagaggucagcgugagc.....................................................................   1       1    seq
    ......................................................................Ngagaggucggugugagc....................   1       1    seq
    ......................................................................agagaggucggugugaUc....................   1       1    seq
    ......................................................................agagagguAggugugagc....................   1       1    seq
    ......................................................................Ugagaggucggugugagc....................   1       1    seq
    ......................................................................agagaggucggugugagU....................   2       1    seq
    ......................................................................Ggagaggucggugugagc....................   113     1    seq
    ......................................................................agagaggucggugugagc....................   580     0    seq
    ......................................................................agagaggucggCgugagc....................   3       1    seq
    ......................................................................agagaggucAgugugagc....................   192     1    seq
```

Provisional ID	:	chr4_11400
Score total	:	1.1
Score for star read(s)	:	-1.3
Score for read counts	:	0
Score for mfe	:	0.8
Score for randfold	:	1.6
Score for cons. seed	:	
Total read count	:	34
Mature read count	:	34
Loop read count	:	0
Star read count	:	0

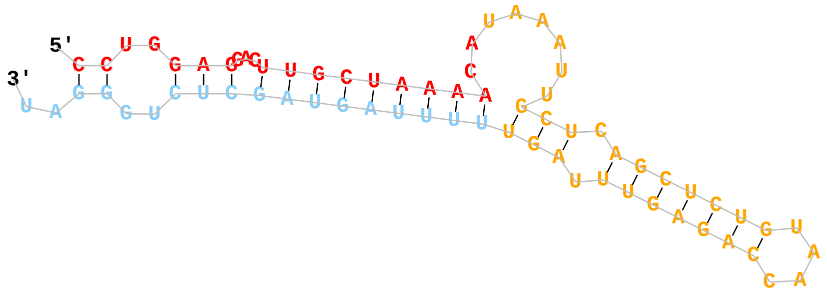

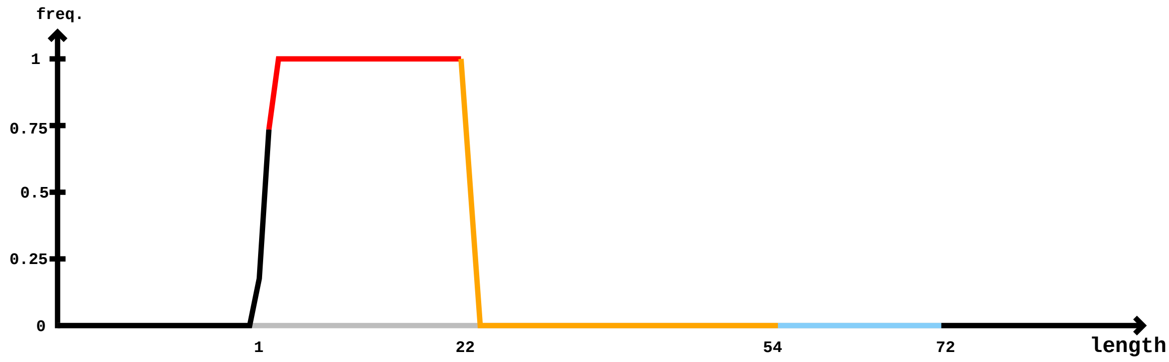


```
                                    Mature                                            Star
5'- uuaaaccagaaguucagccaccuggaggacuugcuaaaacauaaauugcucagcucuguaaccagaguuuaguuuuuaguagcucugggaugaggcuugagaauuuacaug   -3'    exp
    .........((((((((((.((..(((...(((((((((........(((.(((((((....))))))).)))))))))))))))..))....))))...)))))).....    reads    mm    sample
    ...................accuggaggacuugcuaaaaca......................................................................    6        0     seq
    ....................ccuggaggacuugcuaaaaca......................................................................    19       0     seq
    .....................cuggaggacuugcuaaaaca......................................................................    9        0     seq
```

Provisional ID	:	chr8_23042
Score total	:	1.9
Score for star read(s)	:	-1.3
Score for read counts	:	0
Score for mfe	:	1.6
Score for randfold	:	1.6
Score for cons. seed	:	
Total read count	:	113
Mature read count	:	113
Loop read count	:	0
Star read count	:	0

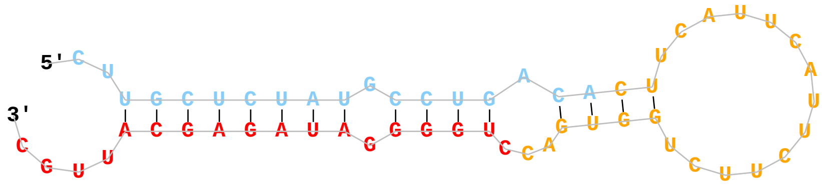

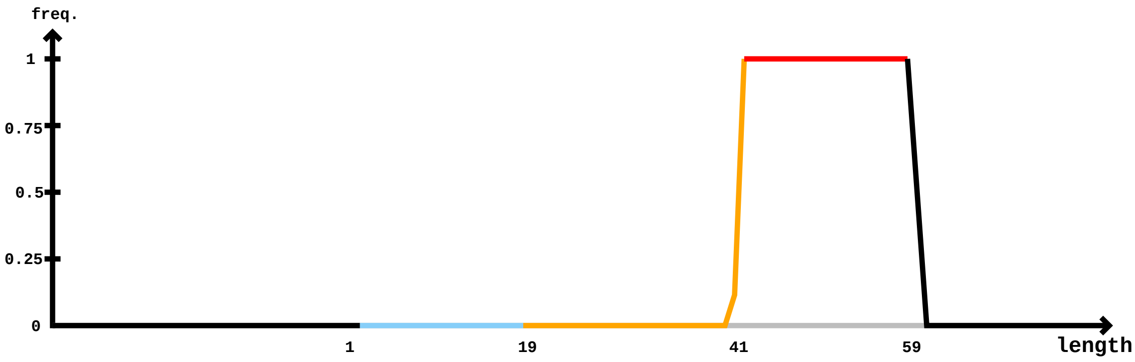


```
                                   Star                                        Mature
5'- aaaggugguggcccaugggucccacucucucuugcucuaugccugacacuucauucauucuucuggugaccuggggauagagcauugcccucccgacucauggcgccc   -3'    exp
    .........((((((((((((...........((((((((.((((.((((..............))))...)))).))))))))..........))))))))).))).   reads      mm         sample
    .....................................................................Gcuggggauagagcauugc....................   13         1          seq
    ......................................................................Guggggauagagcauugc....................   1          1          seq
    ......................................................................cuggggauagagcauugc....................   52         0          seq
    ......................................................................cuggggauagagcauugA....................   1          1          seq
    ......................................................................cuggggauagagcauugcA...................   46         1          seq
```

Provisional ID	:	chr10_28164
Score total	:	1.2
Score for star read(s)	:	-1.3
Score for read counts	:	0
Score for mfe	:	0.9
Score for randfold	:	1.6
Score for cons. seed	:	
Total read count	:	265965
Mature read count	:	265965
Loop read count	:	0
Star read count	:	0

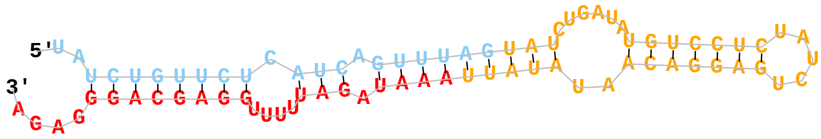

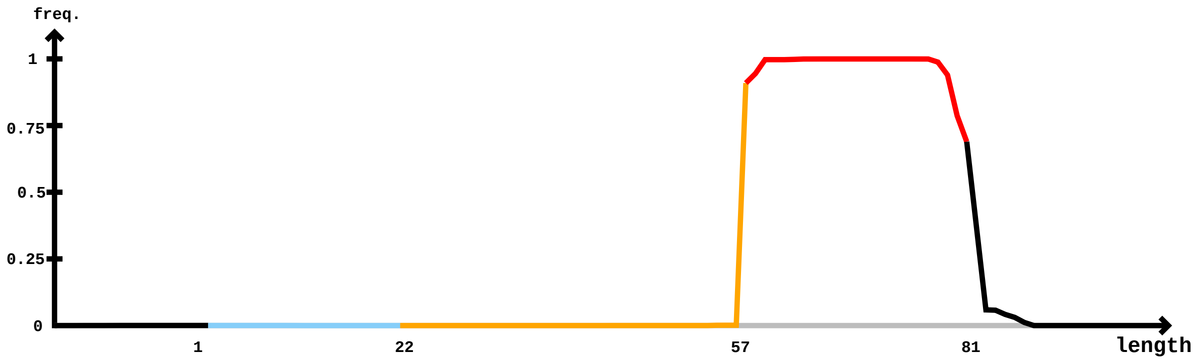

Star Mature

```
5'- aagaucaaguguaguaucuguucucaucaguuuaguaucugauauguccucuaucugaggacaauauauuaaauagauuuuuggagcagggagauggaauaggagcuugcuccg -3'   exp
    ...............((((((((.(((.(((((((((......(((((((.....)))))))..))))))))).)))....))))))))............((((....)))).   reads   mm   sample
    Uagaucaaguguaguaucu...............................................................................................   2       1    seq
    Uagaucaaguguaguaucuguucu..........................................................................................   4       1    seq
    .agaucaaguguaguaucuguuc...........................................................................................   8       0    seq
    .agaucaaguguaguaucuguucu..........................................................................................   6       0    seq
    ..gaucaaguguaguaucug..............................................................................................   10      0    seq
    ..gaucaaguguaguaucuguucu..........................................................................................   6       0    seq
    ....ucaaguguaguaucuguucu..........................................................................................   20      0    seq
    .....caaguguaguaucuguucu..........................................................................................   27      0    seq
    .........guaguaucuguucucaucaguuua.................................................................................   3       0    seq
    ....................................................aucugaggacaauauauuaaauGgauuuuuggagcaggg.......................   1       1    seq
    ....................................................aucugaggacaauauauuaaauGgauuuuuggagcaggga......................   5       1    seq
    ........................................................gaggacaauauauuaaauGgauuuuuggagcaggg.......................   9       1    seq
    ................................................................uauauuaaauGgauuuuuggagcagggaga....................   5       1    seq
    ..................................................................uauuaaauGgauuuuuggagcagggaga....................   2       1    seq
    ..................................................................uauuaaauGgauuuuuggagcagggagaug..................   4       1    seq
    ...................................................................auuaaauGgauuuuuggagcagg........................   9       1    seq
    ...................................................................auuaaauagauuuuuggagcagggag.....................   10      0    seq
    ...................................................................auuaaauGgauuuuuggagcagggag.....................   15      1    seq
    ...................................................................auuaaauGgauuuuuggagcagggaga....................   169     1    seq
    ...................................................................auuaaauGgauuuuuggagcagggagau...................   62      1    seq
    ...................................................................auuaaauGgauuuuuggagcagggagaugga................   2       1    seq
    ...................................................................auuaaauGgauuuuuggagcagggagauggaa...............   6       1    seq
    ...................................................................auuaaauGgauuuuuggagcagggagauggaau..............   8       1    seq
    ....................................................................uuaaauGgauuuuuggagcagggaga....................   3       1    seq
    ....................................................................uuaaauGgauuuuuggagcagggagau...................   12      1    seq
    .....................................................................uaaauGgauuuuuggagcagggagaugg.................   13      1    seq
    ......................................................................aaauGgauuuuuggagca..........................   14      1    seq
    ......................................................................aaauGgauuuuuggagcag.........................   38      1    seq
    ......................................................................aaauGgauuuuuggagcagg........................   3118    1    seq
    ......................................................................aaauGgauuuuuggagcaggg.......................   12706   1    seq
    ......................................................................aaauagauuuuuggagcaggg.......................   7       0    seq
    ......................................................................aaauCgauuuuuggagcaggg.......................   1       1    seq
    ......................................................................aaauUgauuuuuggagcaggga......................   6       1    seq
    ......................................................................aaauagauuuuuggagcaggga......................   14      0    seq
```

Star Mature

```
aagaucaaguguaguaucuguucucaucaguuuaguaucugauauguccucuaucugaggacaauauuaaauagauuuuuggagcagggagauggaauaggagcuugcuccg
....................................................................aaauGgauuuuuggagcaggga......................   40119   1   seq
....................................................................aaauCgauuuuuggagcaggga......................   6       1   seq
....................................................................aaauGgauuuuuggagcagggag.....................   24296   1   seq
....................................................................aaauCgauuuuuggagcagggag.....................   3       1   seq
....................................................................aaauagauuuuuggagcagggag.....................   4       0   seq
....................................................................aaauUgauuuuuggagcagggag.....................   2       1   seq
....................................................................aaauUgauuuuuggagcagggaga....................   11      1   seq
....................................................................aaauGgauuuuuggagcagggaga....................   121455  1   seq
....................................................................aaauCgauuuuuggagcagggaga....................   14      1   seq
....................................................................aaauagauuuuuggagcagggaga....................   31      0   seq
....................................................................aaauCgauuuuuggagcagggagau...................   7       1   seq
....................................................................aaauUgauuuuuggagcagggagau...................   2       1   seq
....................................................................aaauagauuuuuggagcagggagau...................   6       0   seq
....................................................................aaauGgauuuuuggagcagggagau...................   27719   1   seq
....................................................................aaauUgauuuuuggagcagggagaug..................   2       1   seq
....................................................................aaauGgauuuuuggagcagggagaug..................   320     1   seq
....................................................................aaauGgauuuuuggagcagggagaugg.................   4093    1   seq
....................................................................aaauUgauuuuuggagcagggagaugg.................   1       1   seq
....................................................................aaauCgauuuuuggagcagggagaugg.................   1       1   seq
....................................................................aaauGgauuuuuggagcagggagaugga................   2902    1   seq
....................................................................aaauCgauuuuuggagcagggagaugga................   1       1   seq
....................................................................aaauUgauuuuuggagcagggagaugga................   1       1   seq
....................................................................aaauGgauuuuuggagcagggagauggaa...............   4826    1   seq
....................................................................aaauagauuuuuggagcagggagauggaau..............   3       0   seq
....................................................................aaauGgauuuuuggagcagggagauggaau..............   2979    1   seq
.....................................................................aauGgauuuuuggagcaggg.......................   139     1   seq
.....................................................................aauGgauuuuuggagcaggga......................   387     1   seq
.....................................................................aauGgauuuuuggagcagggag.....................   780     1   seq
.....................................................................aauGgauuuuuggagcagggaga....................   6343    1   seq
.....................................................................aauUgauuuuuggagcagggaga....................   1       1   seq
.....................................................................aauagauuuuuggagcagggaga....................   1       0   seq
.....................................................................aauCgauuuuuggagcagggaga....................   2       1   seq
.....................................................................aauGgauuuuuggagcagggagau...................   1710    1   seq
.....................................................................aauGgauuuuuggagcagggagaugg.................   58      1   seq
.....................................................................aauGgauuuuuggagcagggagaugga................   37      1   seq
.....................................................................aauGgauuuuuggagcagggagauggaa...............   131     1   seq
.....................................................................aauGgauuuuuggagcagggagauggaau..............   63      1   seq
......................................................................auCgauuuuuggagcagg........................   3       1   seq
......................................................................auGgauuuuuggagcaggg.......................   137     1   seq
......................................................................auGgauuuuuggagcaggga......................   577     1   seq
......................................................................auagauuuuuggagcagggag.....................   1       0   seq
......................................................................auGgauuuuuggagcagggag.....................   1169    1   seq
......................................................................auagauuuuuggagcagggaga....................   2       0   seq
......................................................................auUgauuuuuggagcagggaga....................   1       1   seq
......................................................................auGgauuuuuggagcagggaga....................   9214    1   seq
......................................................................auCgauuuuuggagcagggaga....................   9       1   seq
......................................................................auCgauuuuuggagcagggagau...................   1       1   seq
......................................................................auGgauuuuuggagcagggagau...................   2542    1   seq
......................................................................auGgauuuuuggagcagggagaug..................   7       1   seq
......................................................................auGgauuuuuggagcagggagaugg.................   90      1   seq
......................................................................auGgauuuuuggagcagggagaugga................   48      1   seq
......................................................................auGgauuuuuggagcagggagauggaa...............   142     1   seq
......................................................................auGgauuuuuggagcagggagauggaau..............   69      1   seq
.......................................................................uGgauuuuuggagcagggagauggaau..............   7       1   seq
........................................................................Ggauuuuuggagcagggagauggaa...............   6       1   seq
.........................................................................gauuuuuggagcagggag.....................   53      0   seq
.........................................................................gauuuuuggagcagggGga....................   1       1   seq
.........................................................................gauuuuuggagcaUggaga....................   1       1   seq
.........................................................................gauuuuuggagcagggaga....................   125     0   seq
.........................................................................gauuuuuggagcagggagau...................   50      0   seq
.........................................................................gauuuuuggagcaUggagaugga................   1       1   seq
.........................................................................gauuuuuggagcagggagaugga................   10      0   seq
.........................................................................gauuuuuggagcagggagauggaa...............   18      0   seq
..........................................................................auuuuuggagcagggaga....................   286     0   seq
..........................................................................auuCuuggagcagggaga....................   1       1   seq
..........................................................................auuuuuggagcagggagau...................   85      0   seq
...........................................................................uuuuuggagcagggagau...................   38      0   seq
...........................................................................uuuuuggagcagggagauggaau..............   6       0   seq
............................................................................uuuuggagcagggagauggaau..............   6       0   seq
.............................................................................uuuggagcagggagaugga................   4       0   seq
```

Star Mature

```
aagaucaaguguaguaucuguucucaucaguuuaguaucugauauguccucuaucugaggacaauauauuaaauagauuuuuggagcagggagauggaauaggagcuugcuccg
...............................................................................uuuggagcagggagauggaauaggagc........     3     0     seq
```

Provisional ID	:	chr12_31893
Score total	:	1.5
Score for star read(s)	:	-1.3
Score for read counts	:	0
Score for mfe	:	1.2
Score for randfold	:	1.6
Score for cons. seed	:	
Total read count	:	147
Mature read count	:	147
Loop read count	:	0
Star read count	:	0

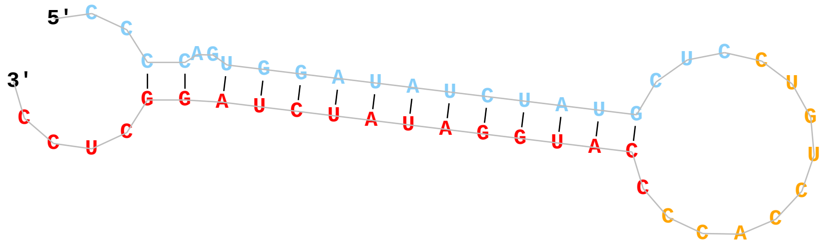

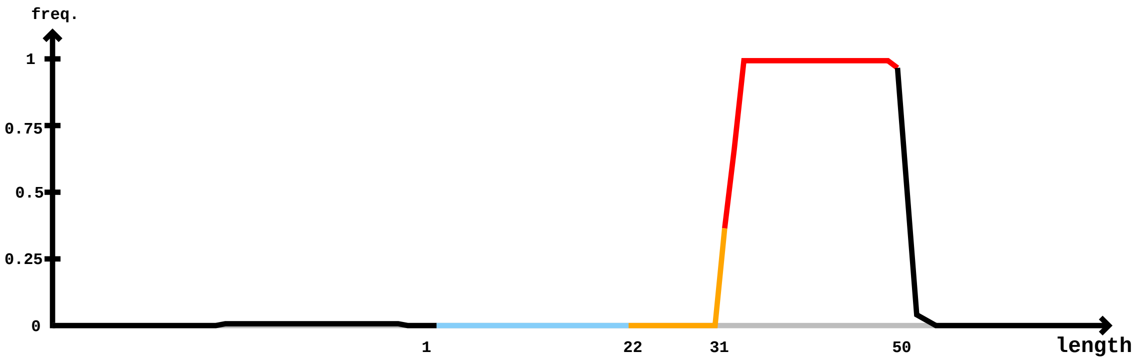

Star Mature

```
5'- ccuccuguccaccccaguggauaucuaggcuccugccaccccaguggauaucuaugcuccuguccaccccauggauaucuaggcuccucuccacccgaguggauaucu   -3'    exp
    .....(((((((.(..(((((......(((....)))...((..((((((((((((.............))))))))))))))......)))))..).)))))))...    reads      mm         sample
    ...............guggauaucuUggcuccug..........................................................................    1          1          seq
    ....................................................................cGauggauaucuaggcuc......................    4          1          seq
    ....................................................................cGauggauaucuaggcucc.....................    40         1          seq
    ....................................................................cGauggauaucuaggcuccu....................    10         1          seq
    .....................................................................Gauggauaucuaggcucc.....................    30         1          seq
    .....................................................................Gauggauaucuaggcuccu....................    14         1          seq
    ......................................................................auggauaucuaggcuccu....................    34         0          seq
    ......................................................................augUauaucuaggcuccu....................    9          1          seq
    ......................................................................auggauaucuaggcuccuA...................    3          1          seq
    ......................................................................auggauaucuaggcuccuAu..................    3          1          seq
```

Provisional ID	:	chr14_35728
Score total	:	2.2
Score for star read(s)	:	-1.3
Score for read counts	:	0
Score for mfe	:	1.9
Score for randfold	:	1.6
Score for cons. seed	:	
Total read count	:	45
Mature read count	:	45
Loop read count	:	0
Star read count	:	0

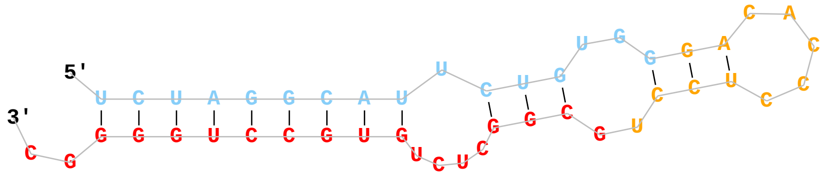

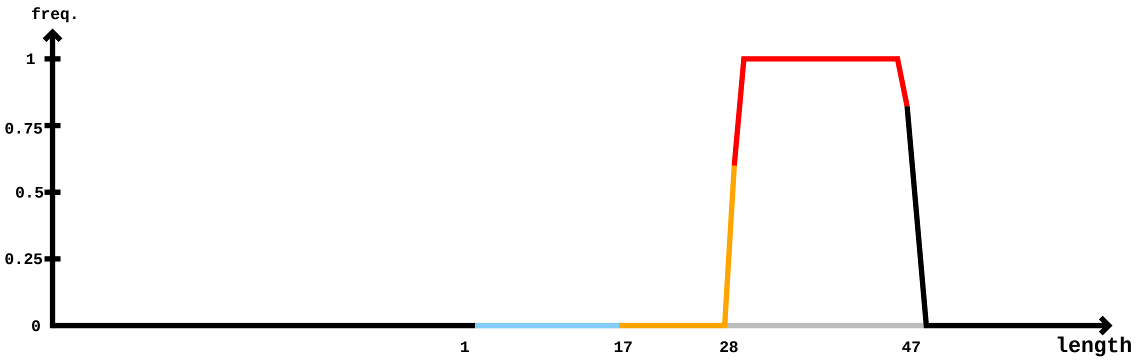

Star Mature

```
5'- agucacccccugccuggcugacuccagguuucucuccucuccucuaggcauucuguggg acacccuccugcggcucugugccuggggcuuggccucauggccccuggc   -3'   exp
    (((((.((.......)).)))))((((.............((((((((((.(((..(((.....)))..)))....))))))))))...((((....))))))))).   reads   mm   sample
    ....................................................................Ucggcucugugccugggg......................   8       1    seq
    ....................................................................Ucggcucugugccuggggc.....................   19      1    seq
    .....................................................................cggcucugugccuggggc.....................   18      0    seq
```

Provisional ID	:	chr16_37655
Score total	:	2.5
Score for star read(s)	:	-1.3
Score for read counts	:	0
Score for mfe	:	2.2
Score for randfold	:	1.6
Score for cons. seed	:	
Total read count	:	64
Mature read count	:	64
Loop read count	:	0
Star read count	:	0

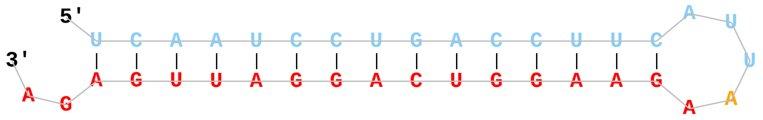

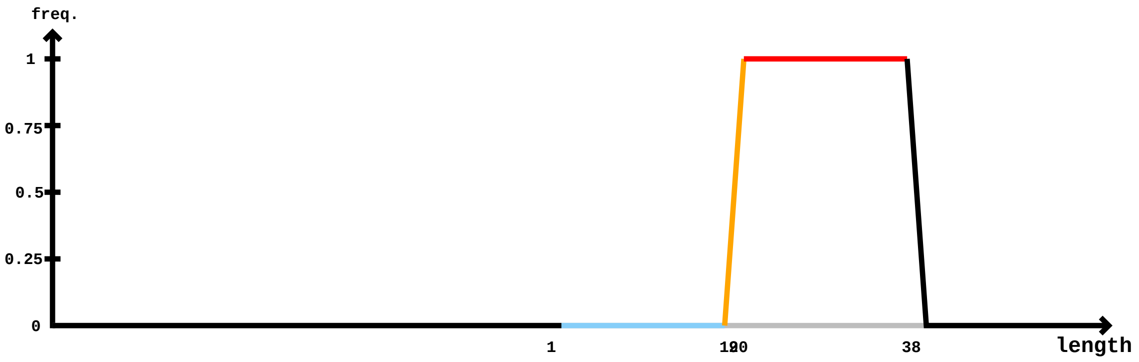


```
                                                                Star                      Mature
5'- cacuccuaccagcuucuugauaacuaccaagaaguucauuagguguuuaucucaauccugaccuucauuaagaaggucaggauugagauuaaaaacccagcagagggu   -3'    exp
    .((.((((..(((((((((........)))))))))...)))).))..((((((((((((((((((.....)))))))))))))))))).....((((......))))    reads      mm        sample
    ......................................................................agaaggucaggauugaga....................    43         0         seq
    ......................................................................agaaggucaggauugagau...................    21         0         seq
```

Provisional ID	:	chr17_39579
Score total	:	1.9
Score for star read(s)	:	-1.3
Score for read counts	:	0
Score for mfe	:	1.6
Score for randfold	:	1.6
Score for cons. seed	:	
Total read count	:	32
Mature read count	:	32
Loop read count	:	0
Star read count	:	0

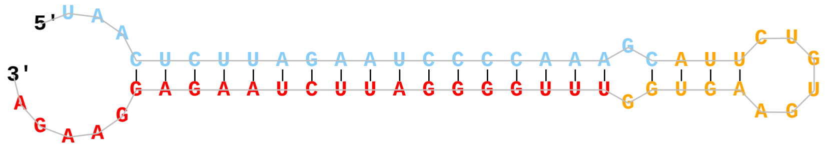

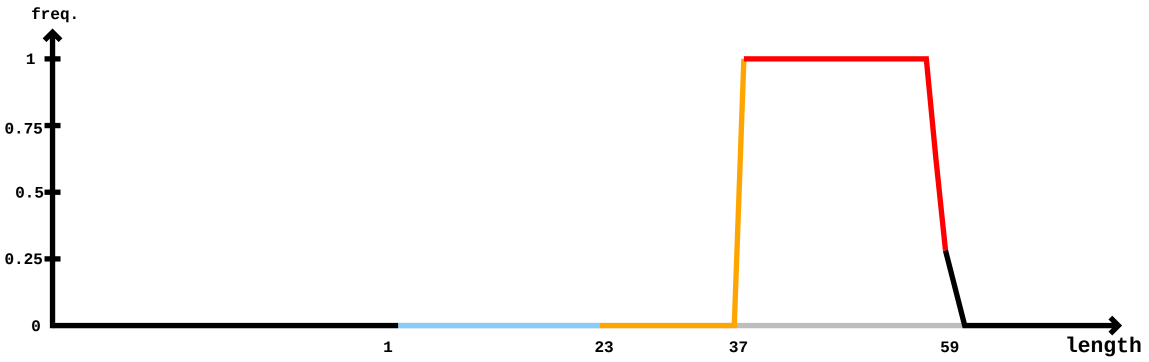


```
                                        Star                                   Mature
5'- aucagcccgaacugcacaaagaacuguacugccuuaacucuuagaauccccaaagcauucugugaagugguuuggggauucuaagaggaagaagaguaaugucauuaau   -3'    exp
    ..(((......)))......((....((((..(((..(((((((((((((((((.((((......)))).)))))))))))))))))....)))))))...))......   reads     mm         sample
    ......................................................................uuuggggauucuaagaggaa...................   4         0          seq
    ......................................................................uuuggggauucuaagaggaU...................   8         1          seq
    ......................................................................uuuggggauucuaagaggaaU..................   6         1          seq
    ......................................................................uuuggggauucuaagaggaag..................   5         0          seq
    ......................................................................uuuggggauucuaagaggaaga.................   9         0          seq
```

Provisional ID	:	chr18_41393
Score total	:	19.5
Score for star read(s)	:	3.9
Score for read counts	:	12.9
Score for mfe	:	1.1
Score for randfold	:	1.6
Score for cons. seed	:	
Total read count	:	37
Mature read count	:	33
Loop read count	:	0
Star read count	:	4

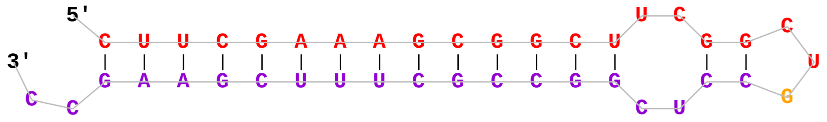

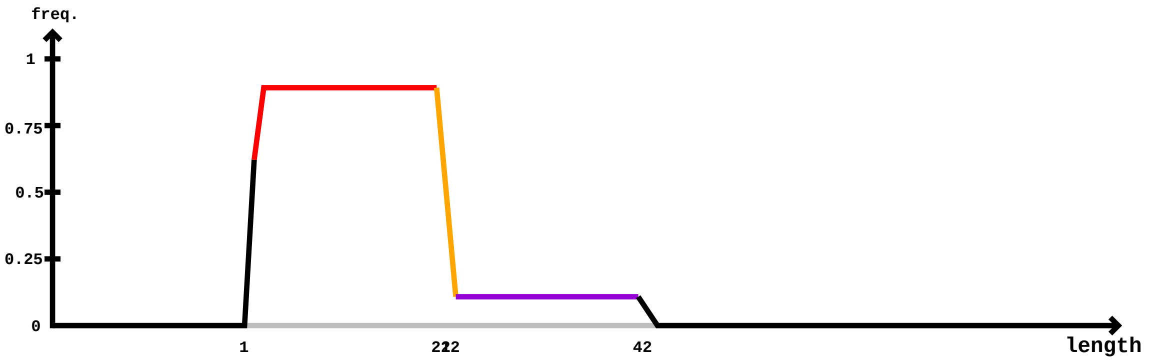

Mature **Star**

```
5'- gagacgcuuuguuugcuaacuucgaaagcggcuucggcugccucggccgcuuucgaagccacuccacaaaacacgccuucggucgguuuaacccuaccucccccccccgc   -3'    obs
    gagacgcuuuguuugcuaacuucgaaagcggcuucggcugccucggccgcuuucgaagccacuccacaaaacacgccuucggucgguuuaacccuaccucccccccccgc          exp
    (((..((.......))...((((((((((((((..((...))..))))))))))))))...)))....((((.((.(....).))))))...................   reads    mm    sample
    ...................cuucgaaagcggcuucggcu.....................................................................   13       0     seq
    ...................cuucgaaagcggcuucggcuU....................................................................   10       1     seq
    ....................uucgaaagcggcuucggcu.....................................................................   10       0     seq
    ........................................ccucggccgcuuucgaagcU................................................   4        1     seq
```

Provisional ID	:	chr18_41488
Score total	:	64.3
Score for star read(s)	:	3.9
Score for read counts	:	68.4
Score for mfe	:	-5.8
Score for randfold	:	-2.2
Score for cons. seed	:	
Total read count	:	146
Mature read count	:	140
Loop read count	:	0
Star read count	:	6

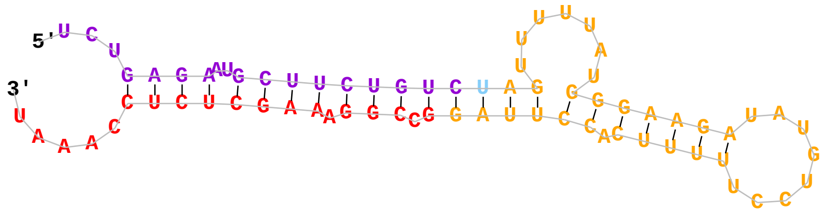

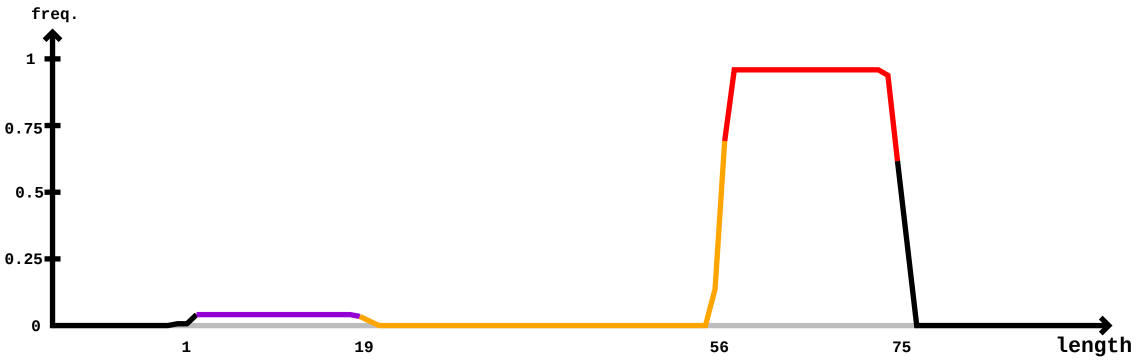

Star   Mature

```
5'- ucacgaguaaguuucugagaaugcuucugucuaguuuuuaugggaagauauguccuuuuucaccuuaggccggaaagcucuccaaauguccacuuacacacacuacaa   -3'   obs
    ucacgaguaaguuucugagaaugcuucugucuaguuuuuaugggaagauauguccuuuuucaccuuaggccggaaagcucuccaaauguccacuuacacacacuacaa         exp
    ......((((((..(.((((..((((((((((((.......(((((((........))))).))))))).))).)))))))).....)...))))))...........   reads   mm   sample
    ...........uuucugagaaugAuucugu..............................................................................   1       1    seq
    .............ucugagaaugcuucuguc.............................................................................   5       0    seq
    ...................................................................ggccggaaagcucuccaa.......................   3       0    seq
    ...................................................................ggccggaaagcucuccaaa......................   13      0    seq
    ...................................................................ggccggaaagcucuccaaaC.....................   4       1    seq
    ....................................................................gccggaaagcucuccaaa......................   34      0    seq
    ....................................................................gccggaaagcucuccaaaC.....................   47      1    seq
    .....................................................................ccggaaagcucuccaaaC.....................   39      1    seq
```
