# Supplementary material for: Systematic evaluation of multiple qPCR platforms, NanoString and miRNA-Seq for microRNA biomarker discovery in human biofluids
Source: Sci Rep. 2021 Feb 24;11:4435. doi: 10.1038/s41598-021-83365-z (PMC7904811; doi:10.1038/s41598-021-83365-z)
Supplement: Supplementary file 5 — Supplementary Information 5. [file 41598_2021_83365_MOESM5_ESM.pdf]

Systematic evaluation of multiple qPCR platforms, NanoString and miRNA-Seq for microRNA biomarker discovery in human biofluids

Lewis Z. Hong[1*], Lihan Zhou[2], Ruiyang Zou[2], Chin Meng Khoo[3], Adeline Lai San Chew[1], Chih-Liang Chin[1], Shian-Jiun Shih[1]

Supplementary Table 1 | Concordance correlation coefficient of Ref. Serum libraries

	5µl_11cyc	5µl_15cyc	5µl_18cyc	10µl_11cyc
5µl_11cyc		0.999	0.999	0.995
5µl_15cyc			0.998	0.996
5µl_18cyc				0.997
10µl_11cyc				

Reproducibility of microRNA expression measurements from miRNA-Seq libraries generated using different input volume of Ref. Serum and/or number of PCR cycles.

Supplementary Table 2 | miRNAs detected above LLOQ – overlap between platforms

	ABI	Exiqon	MiRXES	miRNA-Seq	NanoString
ABI	179	133	143	142	43
Exiqon	133	208	164	167	48
MiRXES	143	164	438	243	55
miRNA-Seq	142	167	243	372	55
NanoString	43	48	55	55	84

Supplementary Table 3 | Novel microRNAs detected by miRNA-Seq in Ref. Serum and selected for validation

miRNA	chr	start[1]	stop[1]	read count[2]	mirDeep2 score[3]
chr1_3503-3p<br>chr1_3503-5p	1	175968397	175968459	461	228.7
chr3_8588-5p	3	49020633	49020697	76	2.5
chr3_9012-5p	3	110967207	110967264	72	1.6
chr3_9719-3p	3	198220824	198220890	1272	2.4
chr4_11400-5p	4	37428529	37428600	47	1.1
chr8_23042-3p	8	6475592	6475684	185	1.9
chr10_28164-3p	10	101364919	101364999	342186	17229.5
chr12_31893-3p	12	129972672	129972723	197	1.5
chr14_35728-3p	14	77278467	77278513	54	2.2
chr16_37655-3p	16	9095609	9095648	87	2.5
chr17_39579-3p	17	44266963	44267022	50	17.9
chr18_41393-3p	18	3448119	3448161	69	11.4
chr18_41488-3p	18	19972964	19973040	260	122.4

[1] Genomic coordinates of predicted microRNA precursor.
[2] Average read count observed per library.
[3] Average quality score assigned by mirDeep2.
